# Spectral, Molecular Modeling, and Biological Activity Studies on New Schiff's Base of Acenaphthaquinone Transition Metal Complexes

**DOI:** 10.1155/2021/6674394

**Published:** 2021-03-22

**Authors:** Khlood S. Abou Melha, Gamil A. Al-Hazmi, Ismail Althagafi, Arwa Alharbi, Ali A. Keshk, Fathy Shaaban, Nashwa El-Metwaly

**Affiliations:** ^1^Department of Chemistry, King Khalid University, P.O. Box 9004, Abha, Saudi Arabia; ^2^Department of Chemistry, Taiz University, P.O. Box 82, Taiz, Yemen; ^3^Department of Chemistry, Umm-Al-Qura University, Makkah, Saudi Arabia; ^4^Department of Chemistry, College of Science, University of Tabuk, Tabuk, Saudi Arabia; ^5^Department of Environment and Health Research, Custodian of Two Holy Mosques Institute for Hajj and Umrah Research, Umm-Al-Qura University, Makkah, Saudi Arabia; ^6^Department of Geomagnetic and Geoelectric, National Research Institute of Astronomy and Geophysics, Kafr Al Masallat, Egypt; ^7^Department of Chemistry, Mansoura University, Mansoura, Egypt

## Abstract

The newly synthesized Schiff's base derivative, N-allyl-2-(2-oxoacenaphthylen-1(2H)-ylidene)hydrazine-1-carbothioamide, has been characterized by different spectral techniques. Its reaction with Co(II), Ni(II), and Zn(II) acetate led to the formation of 1 : 1 (M:L) complexes. The IR and NMR spectral data revealed keto-thione form for the free ligand. On chelation with Co(II) and Ni(II), it behaved as mononegative and neutral tridentate via O, N^1^, and S donors, respectively, while it showed neutral bidentate mode via O and N^1^ atoms with Zn(II). The electronic spectra indicated that all the isolated complexes have an octahedral structure. The thermal gravimetric analyses confirmed the suggested formula and the presence of coordinated water molecules. The XRD pattern of the metal complexes showed that both Co(II) and Ni(II) have amorphous nature, while Zn(II) complex has monoclinic crystallinity with an average size of 9.10 nm. DFT modeling of the ligand and complexes supported the proposed structures. The calculated HOMO-LUMO energy gap, ΔE_H-L_, of the ligand complexes was 1.96–2.49 eV range where HAAT < Zn(II) < Ni(II) < Co(II). The antioxidant activity investigation showed that the ligand and Zn(II) complex have high activity than other complexes, 88.5 and 88.6%, respectively. Accordingly, the antitumor activity of isolated compounds was examined against the hepatocellular carcinoma cell line (HepG2), where both HAAT and Zn(II) complex exhibited very strong activity, IC_50_ 6.45 ± 0.25 and 6.39 ± 0.18 *μ*M, respectively.

## 1. Introduction

Schiff bases are a class of compounds that contain azomethine bond, -C=N-, which results from the condensation of active carbonyl compounds with a primary amine. The Schiff bases are very versatile where the most common Schiff bases have NO donor atoms, but in many cases, the oxygen atom may be replaced by sulfur atoms or added to the donor atoms, NS or NSO, respectively [1]. They are widely applied in several fields such as drugs [2], agriculture [3], luminescent materials [4, 5], and metal anticorrosion [6]. Moreover, in inorganic chemistry, Schiff bases are an interesting ligand due to their tendency to form stable complexes with most transition metal ions. The importance of Schiff base complexes has increased as they may serve as models for biologically important species [7, 8].

Thiosemicarbazones, as a subclass of the Schiff bases, are formed by the reaction of ketone or aldehyde and thiosemicarbazide. Thiosemicarbazones and their complexes have noteworthy significance in biological and chemical studies [9–13]. Their multidonor atoms enable chelation with metal ions to form neutral or charged stable and colored complexes. The remarkable biological activities and varied structural properties of thiosemicarbazone metal complexes promoted their application in the development of therapeutic agents [1, 14, 15]. Lately, two compounds exhibited antitumor activity against several human cell lines [16–18]. The thiosemicarbazone biological properties altered by metal ion coordination, e.g., the lipophilicity that controls the penetration rate into the cell, are changed and so reduce the side effects. Furthermore, the complexes may show new bioactivity which is not displayed by the free ligand [19, 20].

Literature survey showed that acenaphthaquinone and its derivatives were widely used as starting and intermediate materials for the production of different compounds that have pharmaceutical importance [21, 22], pesticides, dyes, drugs, and versatile fluorescent chemosensor [23–26]. However, a few reports on acenaphthaquinone thiosemicarbazone derivatives were observed. The cell proliferation inhibition activity on Friend erythroleukemia cells (FLCs) of acenaphthaquinone mono-thiosemicarbazone derivative and its Cu(II), Ni(II), Fe(III), and Zn(II) metal complexes was reported firstly where the ligand showed stronger inhibition than metal complexes but the Zn(II) complex was higher than other metal complexes. The X-ray single crystal of free ligand showed dimer-like structure in which intramolecular and intermolecular hydrogen bonds were formed. The Ni(II) complex crystal structure indicated that it has distorted octahedral geometry and the ligand behaved as in mononegative tridentate fashion via ONS donors [24]. Moreover, the bimetallic Hg(II) and Cd(II) complexes derived from the 4-phenyl acenaphthaquinone-4-phenyl thiosemicarbazone (APTH) spectral data indicated that the ligand coordinated to the metal ion in neutral tetradentate manner via nitrogen atoms of azomethine and N^2^H groups in addition to both oxygen and sulfur atoms. The APTH was employed as a chelating agent in cloud point extraction procedure of trace amounts of Hg(II) and Cd(II) ions from aqueous medium [27]. Furthermore, the acenaphthaquinone-3-(4-benzylpiperidyl)thiosemicarbazone metal complexes with Co(II), Ni(II), Cu(II), and Zn(II) ions were isolated, and their antibacterial activity against Gram negative and positive bacteria was studied where the complexes exhibited better activity than the ligand. The improved activity was explained by means of a drop in the polarity, which favors permeation of the complexes through the lipid layer of the bacterial cell membrane [28]. The acenaphthaquinone bis(thiosemicarbazone) precipitated onto multiwall carbon nanotubes (MWCNTs) and its Zn(II) and Hg(II) complexes were characterized by IR, TGA, XRD, SEM, and TEM techniques. The antibacterial studies of the functionalized MWCNTs against Gram positive and negative bacteria indicated that the MWCNT loaded with complexes exhibited more potent effect than that loaded with ligand only [29]. Finally, the 1 : 2 (M:L) acenaphthaquinone bis(4-allyl thiosemicarbazone) complexes of Ni(II), Cu(II), and Zn(II) were obtained via one-pot synthetic method. The spectral and single crystal X-ray diffraction of the complexes indicated that ligand chelated to metal ion via the azomethine nitrogen and sulfur atom in mononegative bidentate fashion. The Zn(II) complex was found to be intrinsically fluorescent, so its uptake in IGROV and MCF-7 cancer cells was monitored by confocal fluorescence imaging in addition to comparable cytotoxicity to cisplatin against MCF-7 cell line [30].

Therefore, herein, the synthesis, structure, and cytotoxic activity of a new Schiff base derivative of acenaphthaquinone, N-allyl-2-(2-oxoacenaphthylen-1(2H)-ylidene)hydrazine-1-carbothioamide, and its metal complexes were reported.

## 2. Experimental

### 2.1. Materials and Instrumentation

*N*(4)-allyl-thiosemicarbazide (98.0 %), acenaphthaquinone (99.0 %), and metal acetate salts were purchased from Fluka, Aldrich, or Merck companies. The hepatocellular carcinoma cell line (HepG2) and normal liver Chang cells were obtained from VACSERA Company. The MTT and RPMI-1640 medium were procured from Sigma Company and Fetal Bovine serum from GIBCO.

Elemental analyses were carried out on Perkin-Elmer analyzer 2400 (CHNS). The metal content was obtained by standard methods [31]. The FTIR, ^1^H NMR, ESR, and UV-Vis spectra were recorded on ThermoNicolet IS 10, Bruker Ascend 300 MHz, Brucker E 500 at 9.808 GHz, 100 kHz field modulation, and Unicam UV/Vis UV2 spectrometers, respectively. The TG measurement was carried out using Shimadzu model 50 instrument under nitrogen flow (10 cm^3^/min) and 15°C/min heating rate. The mass spectra were recorded on a Thermo-Scientific DSQ II spectrometer. The powder X-ray diffraction spectra of the metal complexes were recorded on Bruker AXS D8 Advance diffractometer (Cu-K*α* radiation of wavelength *λ* = 1.5406 Å source). Magnetic moment measurements were carried out on a Sherwood Scientific magnetic balance. The complexes' molar conductance, 10^−3^ mol/l in DMF, was recorded on Tacussel conductivity bridge CD6NG.

### 2.2. Preparation of N-Allyl-2-(2-oxoacenaphthylen-1(2H)-ylidene)hydrazine-1-carbothioamide (HAAT)

The reaction of N(4)-allyl-thiosemicarbazide (1.31 g, 0.01 mol) with acenaphthaquinone (1.82 g, 0.01 mol) in ethanol was refluxed for 2 hours. On cooling to room temperature, a yellow precipitate was observed, filtered off, recrystallized from ethanol, and dried in a vacuum desiccator over anhydrous calcium chloride.

*HAAT: N-allyl-2-(2-oxoacenaphthylen-1(2H)-ylidene)hydrazine-1-carbothioamide*. Orange, yield 82%, m.p 195 °C, Anal. Calc. % for C_16_H_13_N_3_OS (295.36): C 65.07; H 4.44; N 14.23. Found%: C 65.22; H 4.64; N 14.08. IR (KBr, cm^−1^): 3320, 3265, 1688, 1640, 936. UV-Vis (DMF, cm^−1^): 35460, 32890, 28570, 25000, 22830, 21835.

### 2.3. Preparation of Solid Complexes

An aqueous solution of metals acetate (1 mmol) was added dropwise to ligand solution (1 mmol, in EtOH). The reaction mixture was refluxed for two hours where the resulting solid complexes were filtered off while being hot, washed successfully with absolute ethanol and diethylether, and finally dried in a desiccator over anhydrous calcium chloride.

*[Co(AAT) (OAc) (EtOH)]*. Dark brown, yield 70%, m.p 210 °C, Anal. Calc. % for C_20_H_21_N_3_O_4_SCo (458.40): C 52.40; H 4.62; N 9.17; M 12.86. Found%: C 52.12; H 4.38; N 8.97; M 12.73. Λ_m_ (DMSO Ω^−1^·cm^2^·mol^−1^) 9.8. IR (KBr, cm^−1^): 3314, 1725, 1703, 1664, 1586, 890. UV-Vis (DMF, cm^−1^): 34970, 32680, 27780, 19160, 17010, 15085.

*[Ni(HAAT) (OAc)*_*2*_ *(H*_*2*_ *O)]*. Brown, yield 73%, m.p 220°C, Anal. Calc. % for C_20_H_21_N_3_O_6_SNi (490.16): C 49.01; H 4.32; N 8.57; M 11.97. Found%: C 48.74; H 4.11; N 8.33; M 12.01. Λ_m_ (DMSO Ω^−1^·cm^2^·mol^−1^) 10.2. IR (KBr, cm^−1^): 3420, 3336, 3279, 1702, 1652, 1619, 930. UV-Vis (DMF, cm^−1^): 34965, 32260, 27935, 20160, 19050, 14750.

*[Zn(HAAT) (OAc)*_*2*_ *]*. Brownish yellow, yield 86%, m.p 230°C, Anal. Calc. % for C_20_H_19_N_3_O_5_SZn (478.38): C 50.17; H 4.00; N 8.78; M 13.65. Found%: C 49.93; H 3.89; N 8.45; M 13.39. Λ_m_ (DMSO Ω^−1^·cm^2^·mol^−1^) 10.3. IR (KBr, cm^−1^): 3323, 3264, 1722, 1677, 1656, 1568, 935. UV-Vis (DMF, cm^−1^): 35210, 32465, 27930, 24390, 21740, 20750.

### 2.4. Molecular Modeling Method

The ligand and complexes' geometry optimization was performed via the Gaussian 09W suite program [32] at DFT/B3LYP level [33–35] and 6–311++G (d,p) basis set. The HOMO-LUMO orbits were demonstrated by the GaussView program [36].

### 2.5. Biological Applications

#### 2.5.1. Antioxidant Activity Screening

The antioxidant activity of the ligand and its complexes was carried out using the ABTS assay procedure [37, 38] in which the 2,2'-azino-bis(3-ethylbenzthiazoline-6-sulfonic acid) (ABTS) and L-ascorbic acid serve as free radical source and standard antioxidant, respectively.

#### 2.5.2. In Vitro Antitumor Activity

The hepatocellular carcinoma cell line (HepG2) was used to investigate the anticancer activity by the well-known MTT assay established on the change in color from yellow to purple due to the conversion of tetrazolium bromide (MTT) to formazan derivative in viable cells, and the relative cell viability % was calculated [39, 40]. The RPMI-1640 with 10% fetal bovine serum was utilized as a medium for the culture of the HepG2.

## 3. Results and Discussion

The reaction of HAAT with the Co(II), Ni(II), and Zn(II) acetate led to the formation of 1 : 1 (M:L) complexes (Table 1). All the isolated solid complexes have nonelectrolytic nature where their molar conductance was 9.8–10.3 Ω^−1^·cm^2^·mol^−1^ [41]. All isolated solid complexes are soluble only in DMF and DMSO.

### 3.1. IR Spectra

**Figure 1:**
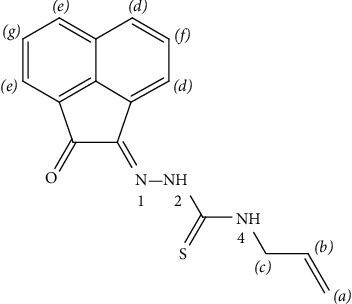
Structure of HAAT.

The HAAT infrared spectrum, in comparison with acenaphthaquinone, presented two new bands at 3320 and 3265 cm^−1^ attributed to *ν*(N^4^H) and *ν*(N^2^H) [27, 42] vibrations (Figure S1), respectively. Moreover, a sharp band at 1688 cm^−1^ was assigned to *ν*(C=O) [43] in addition to a new one at 1640 cm^−1^ attributed to *ν*(C=N^1^) vibration [43] (Figure 1). Furthermore, the new bands observed at 1525, 1453, 1275, and 936 cm^−1^ were assigned to thioamide I, II, III, and *ν*(C=S) [27,44], respectively. The bands observed at 3048, 3029, 2969, and 2955 cm^−1^ were attributed to *ν*_s_(CH) and *ν*_as_(CH) [44] vibrations of aromatic and allyl moieties (Table 2), respectively.

**Figure 2:**
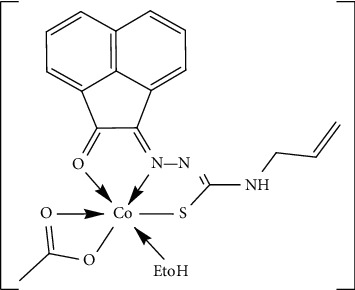
Suggested structure of Co(II) complex.

The comparison between ligand and [Co(AAT) (OAc) (H_2_O)] spectral data revealed that HAAT chelated with the metal ion in a mononegative tridentate fashion *via* the carbonyl oxygen, azomethine nitrogen, and sulfur atom of deprotonated thiol (SH) (Figure 2) where we have the following:

- Only one band centered at 3314 cm^−1^ due to *ν*(N^4^H) vibrations [45] was observed
- The disappearance of vibrational bands due to the *ν*(N^2^H) and *ν*(C=S) along with the appearance of two new bands at 1622 and 890 cm^−1^ attributed to the newly formed azomethine *ν*(C=N^2^) [42, 45] and *ν*(C-S) [45, 46] designated that the ligand transformed into thiol form. Meanwhile, the absence of *ν*(SH) vibrational band, 2500–2600 cm^−1^ range, endorsed that the newly formed SH group deprotonated when the ligand reacted with the metal ion [42, 43] (Figure S2). Both thioamide nitrogen atoms N^2^ and N^4^ were subjected to electron-withdrawing inductive effect with possible delocalization from the thione carbon atom, poorer in electron density. But the azomethine nitrogen atom N^1^ exerted electron release inductive effect without delocalization possibility on the adjacent N^2^ which led to the fact that the N^2^ restored some of its electron density and thus became more suitable for involvement in the enolization process [47]
- The shift to the higher wavenumber of both *ν*(C=O) and *ν*(C=N^1^) vibrational bands, observed at 1703 and 1664 cm^−1^, respectively, revealed their involvement in coordination with the metal ion
- Finally, new bands at 1725 and 1586 cm^−1^ were attributed to *ν*_as_(OAc) and *ν*_s_(OAc) vibrations, respectively, of coordinated acetate ion in bidentate fashion (difference ≈ 139 cm^−1^) [45, 48]. The new bands observed at 530 and 494 cm^−1^ were attributed to *ν*(M-O) in addition to a band at 478 cm^−1^ which was assigned to *ν*(M-N) [45, 49]

**Figure 3:**
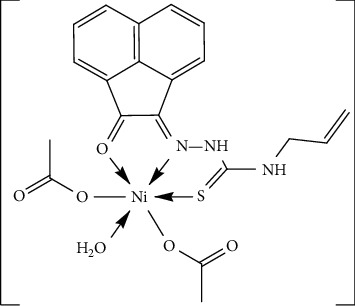
Suggested structure of Ni(II) complex.

The IR spectral data of [Ni(HAAT) (OAc)_2_(H_2_O)] complex displayed two bands at 3336 and 3279 cm^−1^, assigned to the *ν*(N^4^H) and *ν*(N^2^H) vibrations [43, 45], respectively (Table 2). Furthermore, the spectrum exhibited three bands at 1652, 1619, and 930 cm^−1^ attributed to *ν*(C=O), *ν*(C=N^1^), and *ν*(C=S) [45, 46] vibrations, respectively (Figure S3). Moreover, two new bands were observed at 1702 and 1390 cm^−1^ and were assigned to *ν*_as_(OAc) and *ν*_s_(OAc) vibrations, respectively, of coordinated acetate ion in a monodentate fashion (difference ≈ 312 cm^−1^) [45, 48]. The new bands at 528 and 500 cm^−1^ were attributed to *ν*(M-O) in addition to a band at 477 cm^−1^ which was assigned to *ν*(M-N) [45, 49]. Comparison with the ligand spectral data cleared that the bands due to *ν*(C=O), *ν*(C=N), and *ν*(C=S) vibrations were shifted to lower wavenumber which indicated their participation in coordination with the metal ion. Hence, the ligand chelated with the metal ion as neutral tridentate *via* NOS atoms (Figure 3).

**Figure 4:**
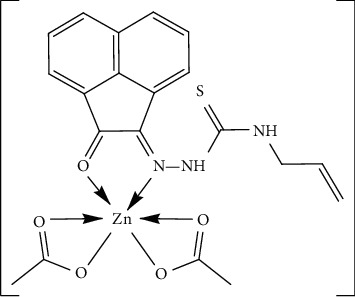
Suggested structure of Zn(II) complex.

Finally, the spectrum of [Zn(HAAT) (OAc)_2_] complex presented *ν*(N^4^H), *ν*(N^2^H), and *ν*(C=S) vibrational bands at 3323, 3264, and 935 cm^−1^ [43, 45], respectively, which are almost at the same position exhibited in ligand spectrum and so endorsed that they are free (Table 2). Additionally, the spectrum showed two bands at 1677 and 1656 cm^−1^ ascribed to *ν*(C=O) and *ν*(C=N^1^) [45, 46] vibrations, respectively. The new bands at 1722 and 1568 cm^−1^ were assigned to *ν*_as_(OAc) and *ν*_s_(OAc) vibrations, respectively, of bidentate acetate ion (difference ≈ 154 cm^−1^) [45, 48]. The new bands observed at 526 and 492 cm^−1^ were attributed to *ν*(M-O) in addition to a band at 482 cm^−1^ which was assigned to *ν*(M-N) [45, 49]. Accordingly, the data revealed that *ν*(C=O) and *ν*(C=N^1^) bands were shifted to lower or higher wavenumber, with respect to the ligand, which designated their participation in coordination with the metal ion as neutral bidentate *via* NO atoms (Figure 4).

### 3.2. ^1^H NMR Spectral Data

**Figure 5:**
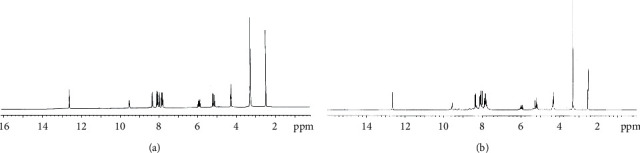
^1^H NMR spectra of HAAT (a) and Zn(II) complex (b) in DMSO-d_6_.

The ligand ^1^H-NMR spectrum, in DMSO-d_6_, displayed singlet signals at 12.65 and 9.55 ppm due to N^4^H and N^2^H protons [27, 44], respectively (Figure 1). As well, to confirm assignment, the addition of D_2_O solution led to the disappearance of these two signals (Figure 5(a)). Furthermore, the spectrum displayed two doublet signals at 8.37 and 8.10 ppm in addition to two multiplet signals at 8.03 and 7.86 ppm assigned to the acenaphthaquinone protons at positions (*d*), (*e*), (*f*), and (*g*) [27, 44], respectively. In addition, three signals were observed at 5.96, 5.19, and 4.31 ppm and were attributed to (CH)_allyl_, (CH_2_)_allyl_, and CH_2_ protons [44, 46], respectively.

The spectrum of Zn(II) complex in d_6_-DMSO, compared to the ligand, displayed the singlet signals of both N^4^H and N^2^H at 12.65 and 9.55 ppm [27, 44], the same positions shown in the ligand spectrum, which confirmed that they are free and the ligand existed in a thione form (Figure 5(b)). Additionally, the spectrum showed the acenaphthaquinone protons as doublet signals at 8.38 and 8.11 in addition to two multiplet signals at 7.99 and 7.82 ppm corresponding to the positions (*d*), (*e*), (*f*), and (*g*) [27, 44], respectively. The signals due to (CH)_allyl_, (CH_2_)_allyl_, and CH_2_ protons were observed at 5.95, 5.21, and 4.32 ppm [44, 46], respectively.

### 3.3. Mass Spectra

**Figure 6:**
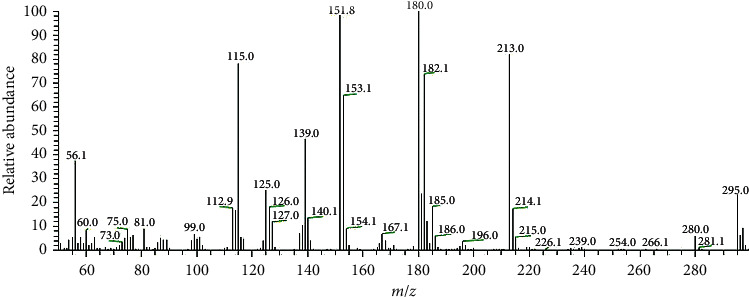
Mass spectrum of HAAT.

**Scheme 1:**
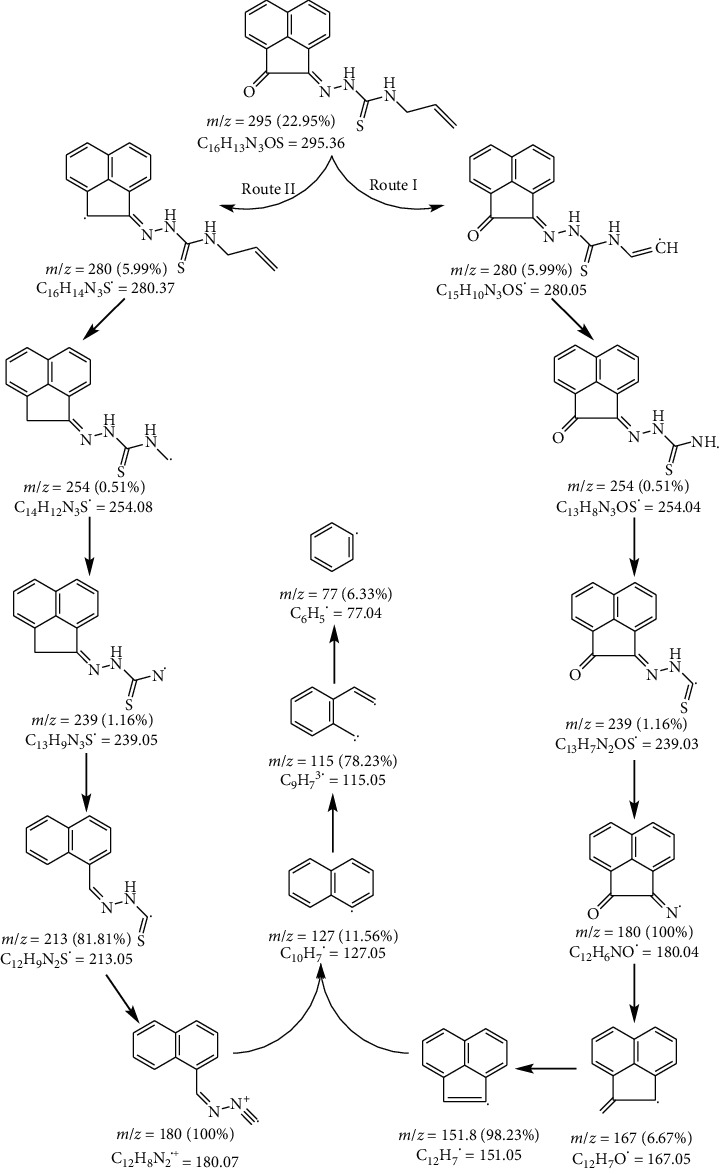
Fragmentation pattern of HAAT.

The HAAT mass spectrum confirmed the proposed molecular formula as it showed a molecular ion peak at *m*/*z* = 295 (22.95 %) that was coincided with its molecular weight (295.36) (Figure 6). The spectrum showed a weak peak at *m*/*z* = 280 (5.99%) which may be due to the loss of methyl radical from the allyl moiety, ^•^CH_3_ (Route I), or oxygen atom of the carbonyl group, ^•^O (Route II), as shown in the fragmentation pattern (Scheme 1). Another peak observed at *m*/*z* = 254 and attributed to C_13_H_8_N_3_OS^•^ and C_14_H_12_N_3_S^•^ formulae in routes I and II, respectively, resulted from the loss of ^•^C_2_H_2_ moiety in both routes. The observed peak at *m*/*z* = 239 was ascribed to loss of ^•^NH and ^•^CH_3_ radicals that led to the formation of C_13_H_7_N_2_OS^•^ and C_13_H_9_N_3_S^•^ moieties (F. Wt. 239.03 and 239.05) in routes I and II, respectively. Furthermore, the spectrum exhibited a base peak at m/*z* = 180 (100%) corresponding to the formula C_12_H_6_NO^•^ (180.04) and C_12_H_8_N_2_^•+^ (180.07) in routes I and II, respectively (Scheme 1).

On the other hand, the Co(II), Ni(II), and Zn(II) complexes' mass spectra showed quite a lot of peaks where the most important one was the molecular ion peak that was observed at 458.37 (2.08%), 490.04 (3.54%), and 478.17 (0.75%), respectively, coincided with the proposed formulae of the complexes (Figures S4–S6). For instance, the Co(II) complex spectrum showed the molecular ion peak at m/*z* = 458 corresponding to the suggested formula [Co(AAT) (OAc) (EtOH)] (M. Wt. = 458.40). The observed peak at *m*/*z* = 412.02 (1.92%) was attributed to the fact that the complex lost ethanol molecule leading to [Co(AAT)(OAc)]^•+^ formula, C_18_H_15_CoN_3_O_3_S^•+^ (F. Wt. = 412.33). The spectrum displayed another peak at *m*/*z* = 368.43 (2.18%) assigned to the C_15_H_7_CoN_3_O_3_S^•+^ formula (F. Wt. = 368.23) that resulted from the degradation of the ligand and acetate ion by losing the C_3_H_8_ fragment.

### 3.4. Electronic Spectra and Magnetic Moment Measurements

The electronic spectrum of the ligand, in DMF, showed two bands at 35460 and 28570 cm^−1^ with a shoulder at 32890 cm^−1^ attributable to the *π* ⟶ *π*^*∗*^ transition of aromatic acenaphthaquinone moiety, carbonyl group, and both of azomethine and thione groups [44], respectively (Table 3). Moreover, the spectrum displayed a broad band at 21835 cm^−1^ with two shoulders at 22830 and 25000 cm^−1^ assigned to the *n* ⟶ *π*^*∗*^ transitions of thione, azomethine, and carbonyl groups [44, 48], respectively (Figure S7(a)).

The Co(II) complex spectrum, in DMF, exhibited a band at 17010 cm^−1^ with a shoulder at 15085 cm^−1^ designated to ^4^T_1g_(F) ⟶ ^4^T_1g_(P) (*υ*_3_) and ^4^T_1g_(F) ⟶ ^4^A_2g_(P) (*υ*_2_) transitions [50], respectively, for octahedral configuration of Co(II) ion (Figure S7(b)). The d^7^-system parameters, *υ*_1_, B, and 10Dq, were calculated *via* the spin allowed transitions, *υ*_3_ and *υ*_2_, and were 7049, 729, and 8020 cm^−1^, respectively, confirming the octahedral arrangement [43, 50]. Furthermore, the spectrum displayed two new bands at 20410 and 19160 cm^−1^ attributed to the charge transfer from ligand to metal (LMCT) and *n* ⟶ *π*^*∗*^ transition of the newly formed azomethine group (C=N^2^) confirming the ligand existence in thiol form (Table 3). The shift of carbonyl and azomethine *n* ⟶ *π*^*∗*^ transition supported its participation in coordination with the metal ion [46, 51]. Finally, the color and magnetic moment value of the Co(II) complex, dark brown and 5.00 B.M., offered further confirmation for the octahedral structure [46, 50, 51].

The Ni(II) complex displayed two bands at 34965 and 27935 cm^−1^ with a shoulder at 32260 cm^−1^ attributed to the intraligand transitions, (*π*⟶*π*^*∗*^)_Acen_, (*π*⟶*π*^*∗*^)_CN&CS_, and (*π*⟶*π*^*∗*^)_CO_ [44, 48], respectively. Moreover, the shoulders observed at 25315, 22525, and 21645 cm^−1^ were assigned to *n*⟶*π*^*∗*^ transitions of the carbonyl, azomethine, and thione groups [44, 48], respectively. The comparison with the ligand spectral data revealed that they were shifted to higher or lower wavenumbers, which confirmed their participation in coordination with the metal ion [50]. Furthermore, a new band was observed at 20160 cm^−1^ with two shoulders at 19050 and 14750 cm^−1^ ascribed to the LMCT, ^3^A_2g_⟶^3^T_1g_(F) (*ν*_2_) and ^3^A_2g_(F)⟶^3^T_2g_(F) (*ν*_1_) transitions, respectively, suggesting that the Ni(II) complex has an octahedral geometry [48, 50]. The spin allowed transitions of the d^8^-system were employed to determine *ν*_3_, B, and Dq parameters and were 31195, 399, and 1476 cm^−1^, respectively. The magnetic moment value was 3.09 B.M. supporting the suggestion of an octahedral structure with ^3^A_2g_ ground term [48, 50].

Finally, the Zn(II) complex displayed two bands at 35210 and 27930 cm^−1^ with a shoulder at 32265 cm^−1^ attributed to the intraligand transitions, (*π*⟶*π*^*∗*^_Acen_, (*π*⟶*π*^∗^)_CN&CS_, and (*π*⟶*π*^*∗*^)_CO_ [44, 48], respectively. Moreover, the band at 20750 cm^−1^ with shoulders at 24390, 22420, and 21740 cm^−1^ was assigned LMCT, (*n*⟶*π*^*∗*^)_CO_, (*n*⟶*π*^*∗*^)_CN_, and (*n*⟶*π*^*∗*^)_CS_ transitions [44, 48], respectively. The data showed that both carbonyl and azomethine *n*⟶*π*^*∗*^ transition bands were shifted to higher or lower wavenumbers supporting their involvement in the metal ion chelation [50].

### 3.5. Thermal Analyses

The thermogravimetric analyses of the ligand and isolated solid complexes confirmed the existence of H_2_O and/or EtOH molecules whichever outside or inside the chelation sphere.

The thermal analysis curve of HAAT showed two decomposition steps at the 150–380 and 380–715°C range. The first step was attributed to the loss of the thione and allyl groups, C_4_H_6_NS (Found: 34.44%; Calcd.: 34.35%), while the second step corresponded to the complete decomposition of the ligand (Found: 61.35%; Calcd.: 61.63%) leading to a carbon ash residue (Found: 4.21%; Calcd.: 4.12%) (Figure S8).

The Co(II) and Zn(II) complexes' TG curves displayed only two successive decomposition stages over 125–445 and 445–610°C ranges. The first stage of the Co(II) complex was due to the loss of coordinated ethanol molecule and acetate anion (Found: 22.12%; Calcd.: 22.92%) while that of Zn(II) was due to the loss of the acetate anions (Found: 25.01%; Calcd.: 24.66%). The second stage in both complexes was attributed to complete decomposition of the ligand leading to a residue of metallic residue (Found: 12.95 and 13.80%; Calcd.: 12.86 and 13.65%, for Co(II) and Zn(II) complexes, respectively) (Table 4).

Finally, the curve of the Ni(II) complex showed that it has thermal stability up to 155°C at which the first decomposition step was observed and attributed to the loss of the coordinated water molecule and acetate anions (Found: 28.01%; Calcd.: 27.75%). The second step was observed over the 390–455°C range and corresponds to the loss of C_3_H_6_N moiety (Found: 11.78%; Calcd.: 11.44%). The third degradation step was extended from 455°C to 640°C and attributed to complete decomposition of the ligand (Found: 48.90%; Calcd.: 48.84%) leaving a metallic residue of nickel metal (Found: 11.31%; Calcd.: 11.97%) (Figure S9).

### 3.6. Powder XRD Patterns

Unfortunately, several trials to get crystals of the newly synthesized metal complexes suitable for single-crystal studies did not succeed which may originate from the fact that they were soluble only in DMSO and DMF. So, the powder X-ray diffraction patterns of Co(II), Ni(II), and Zn(II) complexes were scanned in the range of 5–80 (*θ*) at wavelength of 1.54 Å to investigate their crystallinity degree. The diffraction patterns of both Co(II) and Ni(II) complexes showed no significant peaks, and the trend of the curves decreases from maximum to minimum intensity indicating the amorphous nature of the complexes [52] (Figure S10).

**Figure 7:**
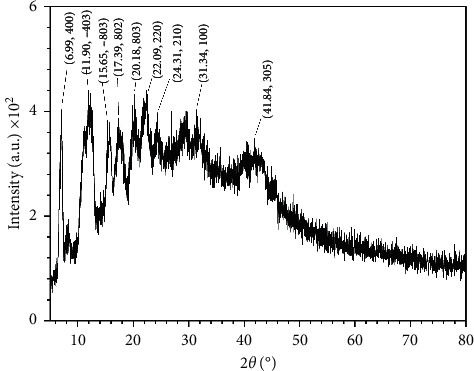
Powder XRD pattern of Zn(II) complex.

On the other hand, the powder X-ray diffraction pattern of [Zn(HAAT) (OAc)_2_] complex showed nine reflections at 2*θ* = 6.99, 11.90, 15.65, 17.39, 20.18, 22.09, 24.31, 31.34, and 41.84° corresponding to interplanar spacing, *d*-values, 12.63, 7.43, 5.66, 5.10, 4.40, 4.02, 3.66, 2.85, and 2.16 Å (Figure 7). Powder XRD peaks were indexed into the face-centered monoclinic and P21/c (14) space group with a lattice constant; *a* = 8.405 ± 0.0003, *b* = 10.183 ± 0.0001, *c* = 13.731 ± 0.0002 Å, *α* = 90°, *β* = 104.4°, and *γ* = 90° [53]. The calculated interplanar spacing, d, together with relative intensities of the most intense peak was recorded and depicted in Table 5. Using the interplanar spacing (*d*) and miller indices (*hkl*), the lattice parameters of monoclinic (2) powder were evaluated from the peak position using the following relation [54, 55]:

(1) $$\begin{array}{c} \frac{1}{d^{2}}=\frac{1}{{\text{sin}}^{2}\beta }\left(\frac{h^{2}}{a^{2}}+\frac{k^{2}{\text{sin}}^{2}\beta }{b^{2}}+\frac{l^{2}}{c^{2}}-\frac{2hl\text{cos}\beta }{ac}\right). \end{array}$$

The crystalline size of the Zn(II) complex, *D*, was evaluated using Debye-Scherrer's equation [56], *D* = 0.9 *λ*/*β*cos*θ*, where *λ* is the X-ray wavelength and *β* is the full width at half maximum intensity (FWHM). The obtained crystalline size was in the range 4.19–16.34 nm exhibiting an average size of 9.10 nm. The microstrain or lattice strain, *ε*, is a measure of the deviation from reference lattice positions and may attribute to the defects and dislocations at the grain boundaries. The lattice strain was determined from the relation, *ε* = *β*/4tan*θ* [57, 58], and was found to be 33.58 × 10^−3^, which denotes high lattice strain. Moreover, the dislocation density, *δ*, which refers to the crystal imperfection, was determined from Williamson and Smallman's relation, *δ* = 1/*D*^2^ [59], and was found to be in the range from 3.76 to 56.95 × 10^−3^ nm with an average value of 24.84 × 10^−3^ nm which reflects the remarkable change of the grain size with values of 2*θ* of the complex.

### 3.7. Optical Band Gap

Tauc's equation was applied to estimate the optical band gap (*E*_*g*_) of the ligand and complexes from their absorption spectra, *αhν* *=* *A (hν -E*_*g*_)^*r*^, where *A* and *r* are independent constants. The *r* value for the indirect transition was 1/2 while, for direct transition, it equals 2 [60, 61]. The intercept with the x-axis, *hν*, in the plot of (*αhν*)^*r*^ against *(hν)* at different *r* values, established by linear portion extrapolating, represents the optical band gap (*E*_*g*_).

**Figure 8:**
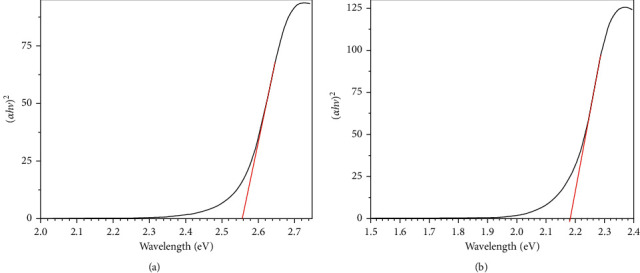
Tauc's plots of HAAT (a) and Ni(II) complex (b).

The plots of the ligand and complexes revealed that the transition mechanism that occurred is a direct one where at *r* = 2, the straight-line was obtained (Figure 8). The data showed that the ligand has a higher *E*_*g*_ value than complexes, 2.56 eV, while the Ni(II) complex has the lowest one, 2.18 eV (Table 6). Therefore, it was concluded that the ligand and complexes have a semiconductive and efficient photovoltaic nature [62–66].

### 3.8. Molecular Modeling

**Figure 9:**
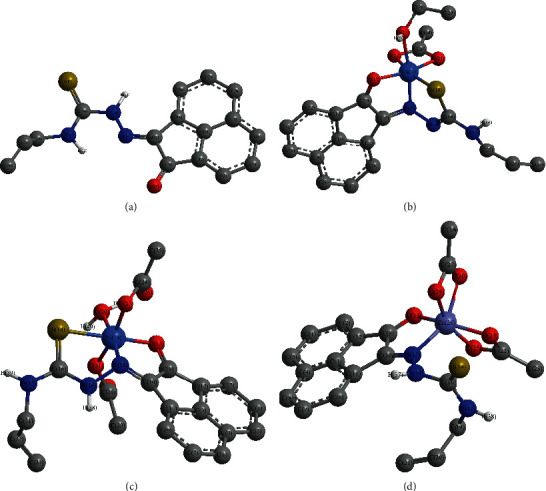
DFT optimized structures of HAAT (a) and Co(II) (b), Ni(II) (c), and Zn(II) (d) complexes.

The HAAT optimized structure has a planar configuration as the dihedral angles data showed, for instance, that both the carbonyl oxygen and azomethine nitrogen atoms were coplanar with each other and the moiety, O-C_nph-o_-C_nph-n_-N^1^ = -0.12° (Table S1). Likewise, the thiosemicarbazone moiety was planar and coplanar with acenaphthaquinone where N^1^-N^2^-CS-N^4^ and N^2^-C_S_-N^4^-C_allyl_ were 0.28 and 179.44°, respectively (Figure 9(a)). The ligand's bond lengths presented almost matched those obtained from single-crystal X-ray data of similar compounds [67], differences in 0.1 to 0.2 Å range (Table S2). The azomethine bond angle, N^2^-N^1^-C_nph-n_, exhibited an ideal value for the *sp*^*2*^ hybridization, 120.57°, while the carbonyl group suffered from small distortion from the standard value, 126.59°. The *sp*^*3*^ hybrid NH groups showed more deviation from the standard value, 109.5°, where, e.g., C_S_-N^2^-N^1^ and C_allyl_-N^4^-C_S_ were 120.39 and 124.04°, respectively (Table S3).

On the other hand, the DFT optimized structure Co(II) complex revealed octahedral geometry around the metal atom in which the ligand has an angular configuration (Figure 9(b)). The dihedral angle data showed that both carbonyl oxygen and azomethine nitrogen were almost planar as O-C_nph-o_-C_nph-n_-N^1^ = -8.73° while the thiocarbonyl carbon, C_s_, and N^2^H nitrogen atoms were tilted on the acenaphthaquinone plane by more than 110° as C_S_-N^2^-N^1^-C_nph-n_ and C_nph-o_-C_nph-n_-N^1^-N^2^ were -112.43 and 131.97°, respectively. The Co(II) atom slightly deviated from the acenaphthaquinone plane where C_nph-n_-C_nph-o_-O-Co and C_nph-o_-C_nph-n_-N^1^-Co angles were 19.27 and -8.08°, respectively. The bond angle data cleared that the complex has a small degree of distortion as, for example, the O-Co-N^1^, S-Co-N^1^, and O-M-S were 92.30, 86.94, and 97.02°, respectively, which are higher or lower than the ideal value (90°). Furthermore, another type of distortion was observed from the bond length data as the Co-S, 2.24 Å, was longer than M-N^1^ and M-O, 1.86 and 1.92 Å, respectively, but compatible with the corresponding X-ray values [28, 67] (Tables S1–S3).

As Co(II) complex, the Ni(II) complex exhibited less bond angle distortion from ideal values of the octahedral structure (Figure 9(c)); e.g., O-Ni-N^1^, S-Ni-N^1^, and O-Ni-S angles were 94.40, 88.31, and 176.93°, respectively. Moreover, the ligand has a less angular configuration, in comparison with the Co(II) complex, where the angle between the carbonyl oxygen and azomethine nitrogen, O-C_nph-o_-C_nph-n_-N^1^, was -5.80° while the thiocarbonyl carbon and N^2^H nitrogen atoms were tilted on the acenaphthaquinone plane as C_S_-N^2^-N^1^-C_nph-n_ and C_nph-o_-C_nph-n_-N^1^-N^2^ were -158.32 and 127.30°, respectively. The Ni(II) atom deviated from the acenaphthaquinone plane where the C_nph-n_-C_nph-o_-O-Ni and C_nph-o_-C_nph-n_-N^1^-Ni angles were 14.34 and -6.51°, respectively. Likewise, the bond lengths exhibited another type of distortion as the Ni-S bond was longer than M-N^1^ and M-O bonds by ∼ 0.5 Å but all were in accordance with the similar complexes reported previously [28, 67] (Tables S1–S3).

Moreover, in Zn(II) complex, the dihedral angles indicated that the carbonyl oxygen, azomethine nitrogen, and N^2^H nitrogen atoms were coplanar to each other and the acenaphthaquinone moiety (Figure 9(d)), where O-C_nph-o_-C_nph-n_-N^1^ = -0.70° and C_nph-o_-C_nph-n_-N^1^-N^2^ = 177.88°. However, the thiocarbonyl carbon atom was tilted on the plane as C_S_-N^2^-N^1^-C_nph-n_ = 154.41°. The Zn(II) atom slightly deviated from the acenaphthaquinone plane where the C_nph-n_-C_nph-o_-O-Zn and C_nph-o_-C_nph-n_-N^1^-Zn angles were 0.15 and 0.82°, respectively. Likewise, the Zn(II) complex exhibited small bond length distortion as Zn-N^1^ was longer than Zn-O bonds by 0.04–0.05 Å and was in agreement with the X-ray reported values [28, 67] (Tables S1–S3).

**Figure 10:**
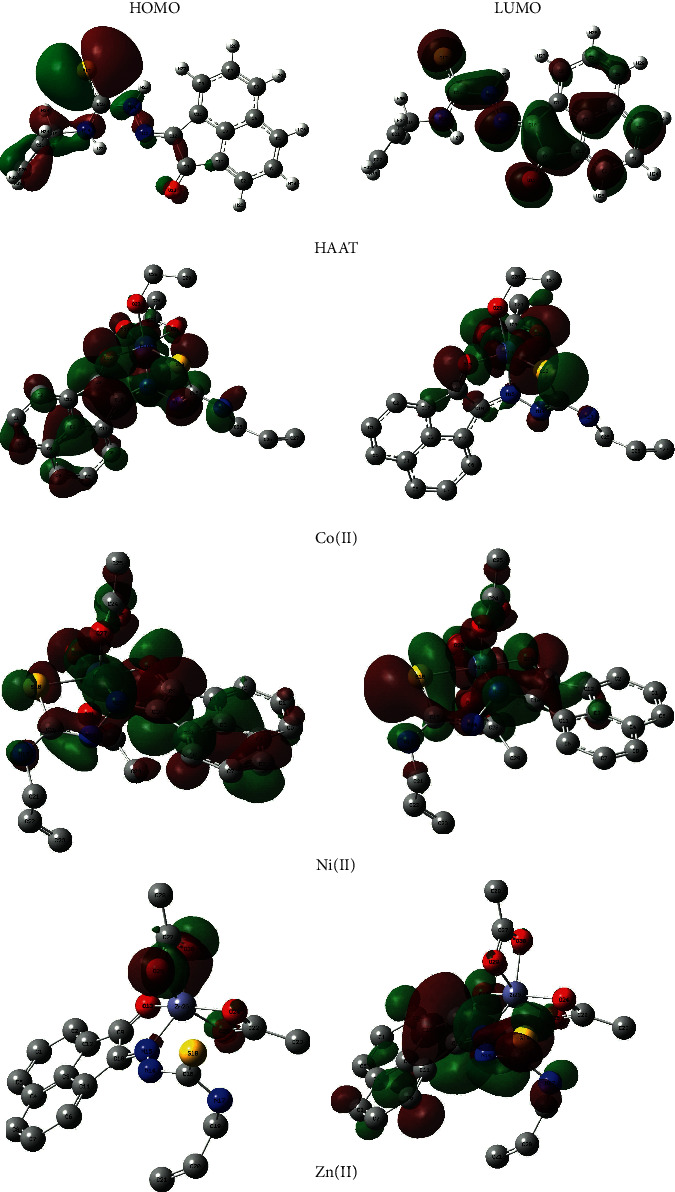
The 3D plots of the investigated compound HOMO and LUMO orbitals.

On the other hand, the energies of the frontier molecular orbitals, HOMO and LUMO, were determined where they act as electron donor and accepter, respectively.  Figure 10 displayed the 3D plots of HOMO and LUMO orbitals of the investigated compounds. As shown in Table 6, the Co(II) complex has the lowest HOMO and LUMO energies (E_H_ and E_L_) while the Zn(II) complex exhibited the highest energies. The chemical stability and intramolecular charge transfer may be correlated with the HOMO-LUMO energy gap, ΔE_H-L_, where the ΔE_H-L_ decrease results in more feasible charge transfer which is one of the significant factors affecting the molecule bioactivity [68–70]. The isolated compounds presented ΔE_H-L_ gap ranging from 1.96 to 2.49 eV and may be ordered as HAAT > Zn(II) > Ni(II) > Co(II) (Table 6). Moreover, it was observed that the ΔE_H-L_ gap was lower than the optical band gap (*Eg*) by only 0.05–0.60 eV.

Finally, the obtained *E*_*H*_ and *E*_*L*_ values were employed to determine some chemical reactivity descriptors like electronegativity (*χ*), global hardness (*η*), softness (*δ*), and electrophilicity (*ω*) using the following equations [68] (Table 6). The data indicated that the Co(II) complex has the highest Lewis acid character and charge transfer resistance, large *χ*, and *η*, respectively. The ligand has the highest electronic acceptability, softest, and electrophilicity that measure the energy reduction that originated from HOMO-LUMO electron flow, high *δ*, and *ω*, respectively.

(2) $$\begin{array}{c} \chi =-\frac{1}{2}\left(E_{HOMO}+E_{LUMO}\right),\\ \eta =-\frac{1}{2}\left(E_{HOMO}-E_{LUMO}\right),\\ \delta =\frac{1}{\eta },\\ \omega =\frac{\chi^{2}}{2\eta }. \end{array}$$

### 3.9. Biological Application

#### 3.9.1. Antioxidant Activity

Antioxidants' scavenge reactive oxygen species (ROS), such as superoxide, hydroxyl, and hydrogen peroxide radicals, cause many life-threatening diseases. Human diseases, like cancer, diabetic mellitus, hypertension, and aging, may be initiated by the ROS capability to destruct DNA, proteins, and membrane functions. The ROS are a by-product of normal metabolism in different subcellular compartments, even under optimal circumstances [71–73].

**Figure 11:**
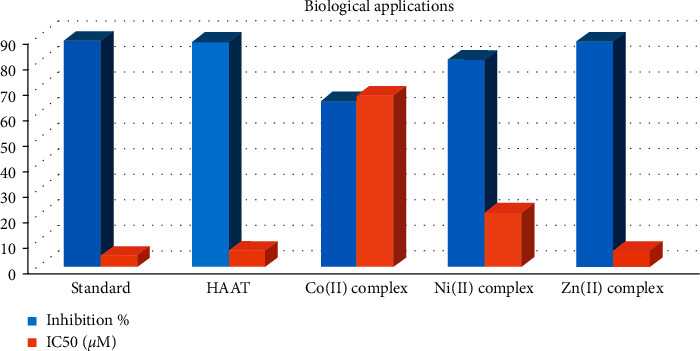
Antioxidant (Inhibition %) and antitumor (IC50) activities of HAAT and its complexes.

The ligand and complexes were examined as an antioxidant by the ABTS method in which L-ascorbic acid was used as a standard material. The data showed that the ligand and Zn(II) complex have high activity than other complexes and close to the ascorbic acid, 88.5 and 88.6%, respectively (Table 7). The Ni(II) complex has comparable activity, 81.4%, while the Co(II) complex was the lowest one, 65.3% (Figure 11). Comparison with previously reported allyl thiosemicarbazide compound, HADTsc [46], indicated that the HAAT and its complexes exhibited slightly higher antioxidant activity than HADTsc.

Finally, the L-ascorbic acid antioxidant activity may originate from its action as a reducing substance; i.e., it donates high-energy electrons to neutralize free radicals [74]. Thus, the activity of ligand may be attributed to its capability to serve as electron donors via heteroatom lone pair of electrons and its low HOMO energy. Moreover, the higher Zn(II) complex activity, in comparison with other complexes, may be attributed to the change in chelation environment around metal ion as all complexes have octahedral geometry but only the Zn(II) complex has higher electron donor ability due to the lone pair of electron of the free sulfur atom as well as lower HOMO energy.

#### 3.9.2. In Vitro Antitumor Activity

**Figure 12:**
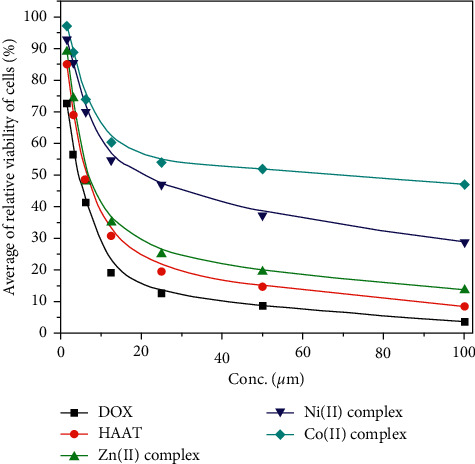
In vitro cytotoxic activities of HAAT and its complexes compared with the standard doxorubicin against HepG2cell line.

The thiosemicarbazones and its complexes presented well-established anticancer activity [75, 76] which stimulated the study of HAAT and its metal complexes' cytotoxic activities against the hepatocellular carcinoma cell line (HepG2), the main type of liver cancer that is the second cause of cancer-related death [77] (Table 7). Like the antioxidant activity, both HAAT and Zn(II) complex exhibited very strong activity, IC_50_ 6.45 ± 0.25 and 6.39 ± 0.18 *μ*M, respectively, comparable to the doxorubicin standard, 4.50 ± 0.2 *μ*M. The Ni(II) complex has moderate activity, IC_50_ 21.46 ± 0.72, while the Co(II) complex has weak cytotoxic activity, weak activity 67.31 ± 1.35 *μ*M (Figure 11). In comparison with the data of allyl thiosemicarbazide compound, HADTsc [46] earlier reported, it was clear that the HAAT and its complexes displayed higher cytotoxic activity than HADTsc. Moreover, the average cells' relative viability percent at different concentrations of examined compounds indicated that ligand has a higher potent effect than the complexes at different concentrations while the Zn(II) complex was the most active complex (Figure 12). The selectivity index (SI) was calculated as the average of the IC_50_ value in the normal cell line divided by the IC_50_ value in the cancer cell line obtained, where the SI > 2 indicates high selectivity [78] (Table 7). Although both HAAT and Zn(II) complex exhibited very strong activity, their SI was 1.14 and 1.28, respectively, which indicated that they processed low selectivity toward the cancer cells. Also, the Co(II) and Ni(II) complexes exhibited lower SI in accordance with their high IC_50_ values.

Finally, the biological activity of metal complexes mainly depends on the coordination sphere around the central metal ion. According to Tweedy's chelation theory, the chelation process leads to a reduction of the metal atom polarity through partial sharing of its positive charge with donor groups and possible electron delocalization over the entire molecule which results in increasing the lipophilic character of the complex. Thus, chelation enhances the penetration of the complexes through the cell membrane and the deactivation of various cellular enzymes metal-binding sites in addition to denaturation of cellular proteins causing the normal cellular processes to be impaired [79–81]. Hence, from the structure point of view, the Zn(II) complex has an octahedral geometry, like other complexes, in which the ligand chelated via the carbonyl oxygen and azomethine nitrogen atoms while the sulfur atom is free in addition to two bidentate acetate ions. Therefore, its activity may originate from (i) higher permeability through the cell membrane as it has more lipophilic character and (ii) the capability to form hydrogen bonds via the free sulfur atom with the active centers of different cellular constituents resulting in interference with normal cellular processes [79–81].

## 4. Conclusion

The spectral characterization of the new Schiff's base derivative, HAAT, N-allyl-2-(2-oxoacenaphthylen-1(2H)-ylidene)hydrazine-1-carbothioamide, revealed that it is in thione form. The HAAT formed 1 : 1 (M:L) octahedral complexes with Co(II), Ni(II), and Zn(II) acetates. The ligand chelated with Co(II), Ni(II), and Zn(II) ions as ONS donor in mononegative and neutral tridentate in Co(II) and Ni(II) complexes, respectively, while in Zn(II) complex, it acts as neutral bidentate, via ON atoms. The DFT calculations showed that the ligand has a planar structure while it has bent conformation reflecting its flexibility. The antioxidant activity investigation revealed that the ligand and Zn(II) complex have high activity almost equal to the standard material, ascorbic acid. Similarly, the MTT assay was utilized for the examination of the antitumor activity using the hepatocellular carcinoma cell line (HepG2). The data revealed that the ligand and Zn(II) complex presented very strong activity.

## Figures and Tables

**Table 1:** Analytical and physical data of HAAT and its complexes.

Compound (mol. formula; Wt.)	Color	m.p. (°C)	Elemental analyses; found (calcd.)				Λ_m_^*∗*^
			C	H	N	M	
HAAT (C_16_H_13_N_3_OS; 295.36)	Orange	195	65.22 (65.07)	4.64 (4.44)	14.08 (14.23)	—	—
[Co(AAT) (OAc) (EtOH)] (C_20_H_21_N_3_O_4_SCo; 458.40)	Dark brown	210	52.12 (52.40)	4.38 (4.62)	8.97 (9.17)	12.73 (12.86)	9.8
[Ni(HAAT) (OAc)_2_(H_2_O)] (C_20_H_21_N_3_O_6_SNi; 490.16)	Brown	220	48.74 (49.01)	4.11 (4.32)	8.33 (8.57)	12.01 (11.97)	10.2
[Zn(HAAT) (OAc)_2_] (C_20_H_19_N_3_O_5_SZn; 478.38)	Brownish yellow	230	49.93 (50.17)	3.89 (4.00)	8.45 (8.78)	13.39 (13.65)	10.3

^*∗*^Measured in DMSO Ω^−1^·cm^2^·mol^−1^.

**Table 2:** Infrared spectral date of HAAT and its metal complexes.

Vibration	HAAT	Complexes		
		Ni(II)	Co(II)	Zn(II)
*ν*(OH)_solv._	**—**	3420	3425	—
*ν*(N^4^H)	3320	3336	3314	3323
*ν*(N^2^H)	3265	3279	—	3264
*ν*(CH)_Ar._	3048, 2955	3070, 2955	3060, 2957	3050, 2958
*ν*(CH)_Allyl_	3029, 2969	3020, 2922	3030, 2918	3018, 2920
*ν*(C=O)_OAc_	—	1702, 1390	1725, 1586	1722, 1568
*ν*(C=O)	1688	1652	1703	1677
*ν*(C=N)	1640	1619	1664	1656
*ν*(C=C)_Ar_	1607	1602	1601	1604
Thioamide I	1525	1520	1513	1524
Thioamide II	1453	1466	1467	1451
Thioamide III	1275	1251	1234	1250
*ν*(N-N)	1146	1172	1117	1175
*ν*(C-O)	1052	1092	1080	1083
*ν*(C=S)	936	930	890 (C-S)	935
*ρ*(NH)	794	775	775	775
*ν*(M-O)	—	528, 500	530, 494	526, 492
*ν*(M-N)	—	477	478	482

**Table 3:** Electronic spectral date of AATH and its metal complexes.

Compound	Transitions	*µ*_eff._
HAAT	35460 (*π⟶π*^*∗*^)_Acen_, 32890 (*π⟶π*^*∗*^)_CO_, 28570 (*π⟶π*^*∗*^)_CN&CS_, 25000 (*n*⟶*π*^*∗*^)_CO_, 22830 (*n*⟶*π*^*∗*^)_CN_, 21835 (*n*⟶*π*^*∗*^)_CS_	—
[Co(AAT) (OAc) (H_2_O)]	34970 (*π⟶π*^*∗*^)_Acen_, 32680 (*π⟶π*^*∗*^)_CO_, 27780 (*π⟶π*^*∗*^)_CN&CS_, 25380 (*n*⟶*π*^*∗*^)_CO_, 22320 (*n*⟶*π*^*∗*^)_CN_, 20410 (*n*⟶*π*^*∗*^)_CN_^2^, 19160 (LMCT), 17010 ^4^T_1g_(F)⟶^4^T_1g_(P) (*υ*_3_), 15085 ^4^T_1g_(F)⟶^4^A_2g_(P) (*υ*_2_)	5.00
[Ni(HAAT) (OAc)_2_(H_2_O)]	34965 (*π⟶π*^*∗*^)_Acen_, 32260 (*π⟶π*^*∗*^)_CO_, 27935 (*π⟶π*^*∗*^)_CN&CS_, 25315 (*n*⟶*π*^*∗*^)_CO_, 22525 (*n*⟶*π*^*∗*^)_CN_, 21645 (*n*⟶*π*^*∗*^)_CS_, 20160 (LMCT), 19050 ^3^A_2g_⟶^3^T_1g_(F) (*ν*_2_), 14750 ^3^A_2g_(F)⟶^3^T_2g_(F) (*ν*_1_)	3.09
[Zn(HAAT) (OAc)_2_]	35210 (*π⟶π*^*∗*^)_Acen_, 32465 (*π⟶π*^*∗*^)_CO_, 27930 (*π⟶π*^*∗*^)_CN&CS_, 24390 (*n*⟶*π*^*∗*^)_CO_, 22420 (*n*⟶*π*^*∗*^)_CN_, 21740 (*n*⟶*π*^*∗*^)_CS_, 20750 (LMCT)	—

**Table 4:** Thermal gravimetric analysis of HAAT and its complexes.

Compound	Temp. range (°C)	Wt. loss %		Fragment lost
		Found	Calcd.	
HAAT	150–380	34.44	34.25	HCNS + CH_3_CH=CH_2_
	380–715	61.35	61.63	C_11_H_6_N_2_O
	Residue	4.21	4.12	Carbon ash

[Co(AAT) (OAc) (H_2_O)]	180–445	22.56	22.93	OAc + EtOH
	445–620	60.93	60.73	C_16_H_12_N_3_S
	Residue	16.51	16.35	CoO

[Ni(HAAT) (OAc)_2_(H_2_O)]	155–390	28.01	27.75	(OAc)_2_ + H_2_O
	390–455	11.68	11.44	C_3_H_6_N
	455–640	45.28	45.56	C_13_H_7_N_2_OS
	Residue	15.03	15.24	NiO

[Zn(HAAT) (OAc)_2_]	125–365	24.87	24.62	(OAc)_2_
	365–620	58.29	58.4	C_16_H_13_N_3_OS
	Residue	16.84	16.99	ZnO

**Table 5:** Powder X-ray data of Zn(II) complex.

2*θ* (°)	*hkl*	*β* (FWHM) (°)	*d* (Å)	*D* (nm)	*ε* ×10^−3^	*δ* (nm) ×10^−3^
6.99	400	0.76	12.63	10.48	54.23	9.10
11.90	−403	1.91	7.43	4.19	79.77	56.95
15.65	−803	1.10	5.66	7.29	34.89	18.79
17.39	802	1.90	5.10	4.22	54.31	56.14
20.18	803	0.53	4.40	15.24	12.98	4.31
22.09	220	0.96	4.02	8.46	21.37	13.96
24.31	210	0.72	3.66	11.31	14.55	7.82
31.34	100	0.50	2.85	16.34	7.85	3.75
41.84	305	1.95	2.16	4.35	22.29	52.78
***Average***	—	***1.15***	***5.32***	***9.10***	***33.58***	***24.84***

**Table 6:** The HOMO energy (E_H_), LUMO energy (E_L_), energy gap (ΔE_H-L_), electronegativity (*χ*), global hardness (*η*), softness (*δ*), electrophilicity (*ω*), and optical band gap (*E*_*g*_) of the ligand and metal complexes in eV.

Compound	E_H_	E_L_	ΔE_H-L_	*χ*	Η	*δ*	*ω*	*E*_*g*_
HAAT	−6.07	−4.11	1.96	5.09	0.98	1.02	13.25	2.56
Co(II)	−6.53	−4.04	2.49	5.29	1.25	0.80	11.21	2.74
Ni(II)	−6.15	−4.02	2.13	5.08	1.06	0.94	12.15	2.18
Zn(II)	−6.01	−3.98	2.03	5.00	1.02	0.98	12.30	2.39

**Table 7:** ABTS antioxidant assay and in vitro cytotoxicity against HepG2 cell line of the ligand and its complexes.

ABTS assay		In vitro cytotoxicity HePG2		
Compounds	Inhibition %	Compounds	IC_50_ (*µ*M)^*∗*^	SI
**L-Ascorbic acid**	88.8	**Doxorubicin**	4.50 ± 0.2	3.28
**HAAT**	88.4	**HAAT**	6.45 ± 0.25	1.14
**Co(II) complex**	65.3	**Co(II) complex**	67.31 ± 1.35	0.82
**Ni(II) complex**	81.4	**Ni(II) complex**	21.46 ± 0.72	0.93
**Zn(II) complex**	88.6	**Zn(II) complex**	6.39 ± 0.18	1.28

^*∗*^IC_50_: 1–10 (very strong), 11–20 (strong), 21–50 (moderate), 51–100 (weak), and above 100 (noncytotoxic).

## Data Availability

The data that support the findings of this study are available in the supplementary material of this article.

## Supplementary Materials

Supplementary Materials Figure S1: IR spectrum of HAAT in comparison with acenaphthaquinone. Figure S2: IR spectrum of Co(II) complex. Figure S3: IR spectrum of Ni(II) complex. Figure S4: The mass spectrum of the Co(II) complex. Figure S5: The mass spectrum of the Ni(II) complex. Figure S6: The mass spectrum of the Zn(II) complex. Figure S7: Electronic spectra of HAAT (a) and Co(II) complex (b). Figure S8: The TG curve of HAAT. Figure S9: The TG curve of Ni(II) complex. Figure S10: The powder XRD pattern of the Co(II) and Ni(II) complexes. Table S1: DFT calculated dihedral angles of the ligand and metal complexes (°). Table S2: DFT calculated bond length of the ligand and metal complexes (Å). Table S3: DFT calculated bond angles of the ligand and metal complexes (°).
