# Supplementary material for: Spectral, Molecular Modeling, and Biological Activity Studies on New Schiff's Base of Acenaphthaquinone Transition Metal Complexes
Source: Bioinorg Chem Appl. 2021 Mar 22;2021:6674394. doi: 10.1155/2021/6674394 (PMC8012121; doi:10.1155/2021/6674394)
Supplement: Supplementary Materials — Figure S1: IR spectrum of HAAT in comparison with acenaphthaquinone. Figure S2: IR spectrum of Co(II) complex. Figure S3: IR spectrum of Ni(II) complex. Figure S4: The mass spectrum of the Co(II) complex. Figure S5: The mass spectrum of the Ni(II) complex. Figure S6: The mass spectrum of the Zn(II) complex. Figure S7: Electronic spectra of HAAT (a) and Co(II) complex (b). Figure S8: The TG curve of HAAT. Figure S9: The TG curve of Ni(II) complex. Figure S10: The powder XRD pattern of the Co(II) and Ni(II) complexes. Table S1: DFT calculated dihedral angles of the ligand and metal complexes (°). Table S2: DFT calculated bond length of the ligand and metal complexes (Å). Table S3: DFT calculated bond angles of the ligand and metal complexes (°). [file 6674394.f1.docx]

**Figure S1**. IR spectrum of HAAT in comparison with acenaphthaquinone.





**Figure S2**. IR spectrum of Co(II) complex.





**Figure S3**. IR spectrum of Ni(II) complex.

**
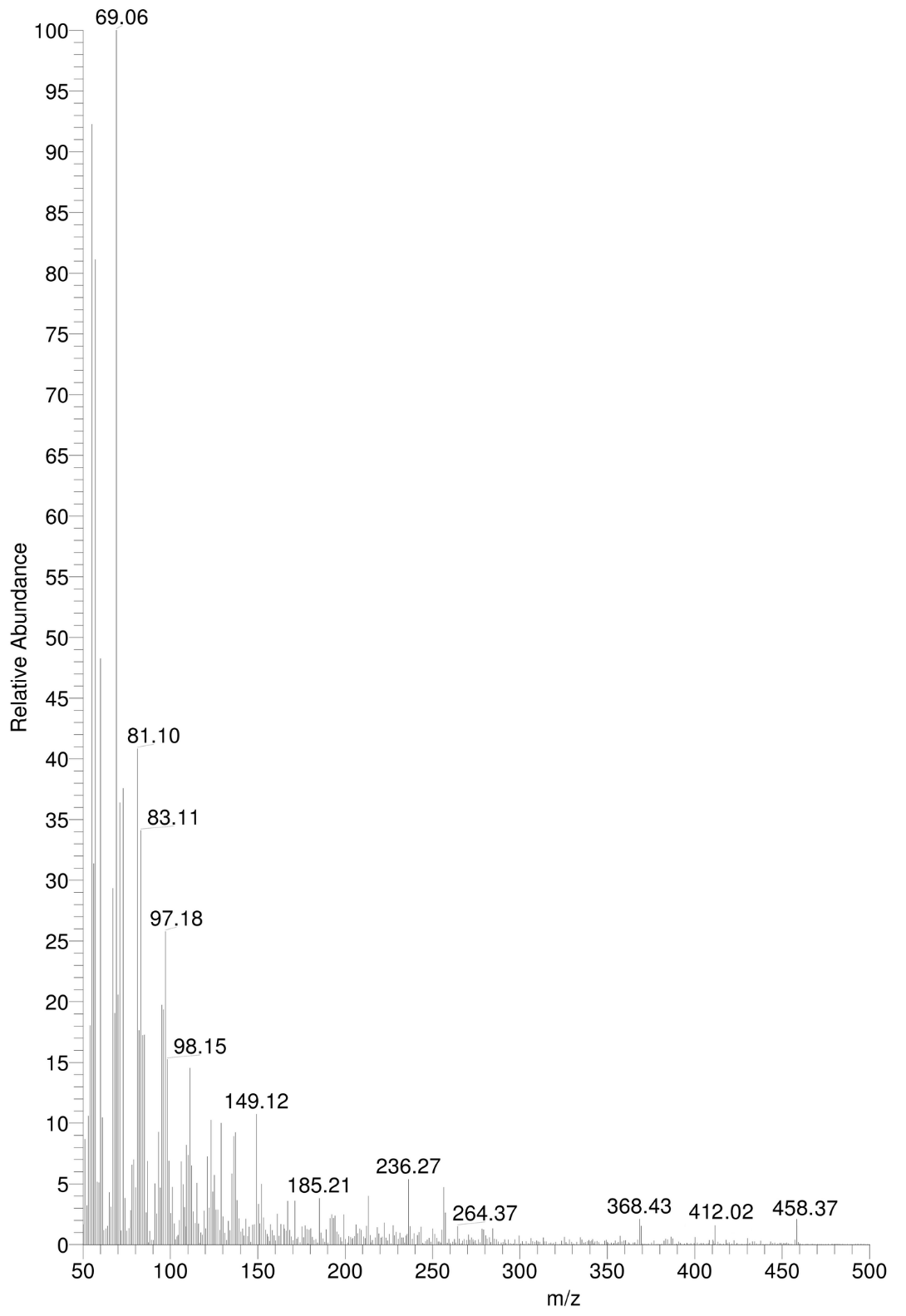
**

**Figure S4.** The Mass spectrum of the Co(II) complex.

**
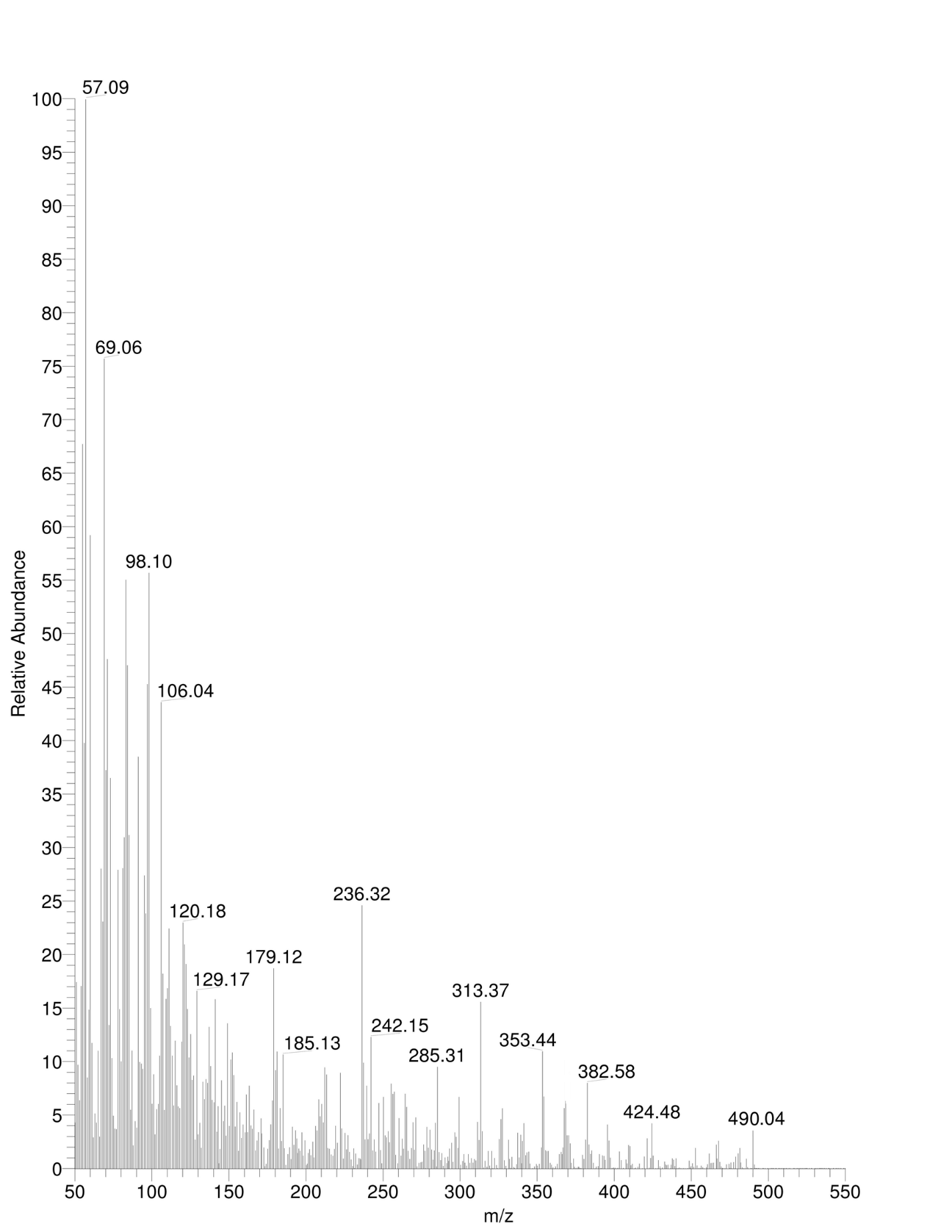
**

**Figure S5.** The Mass spectrum of the Ni(II) complex.

**
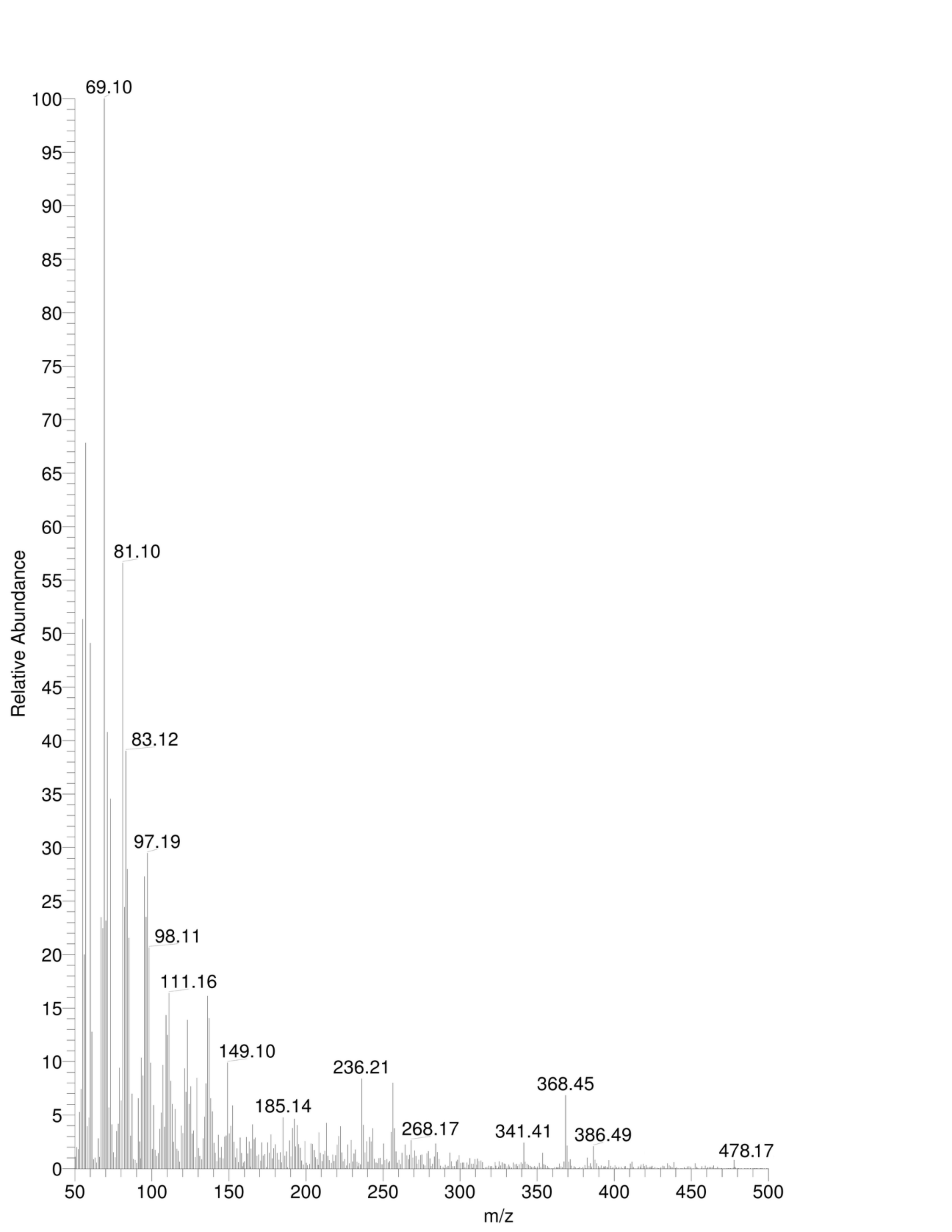
**

**Figure S6.** The Mass spectrum of the Zn(II) complex.

|  |  |
| --- | --- |

**Figure S7**. Electronic spectra of HAAT (A) and Co(II) complex (B).





**Figure S8**. The TG curve of HAAT.





**Figure S9**. The TG curve of Ni(II) complex.

**
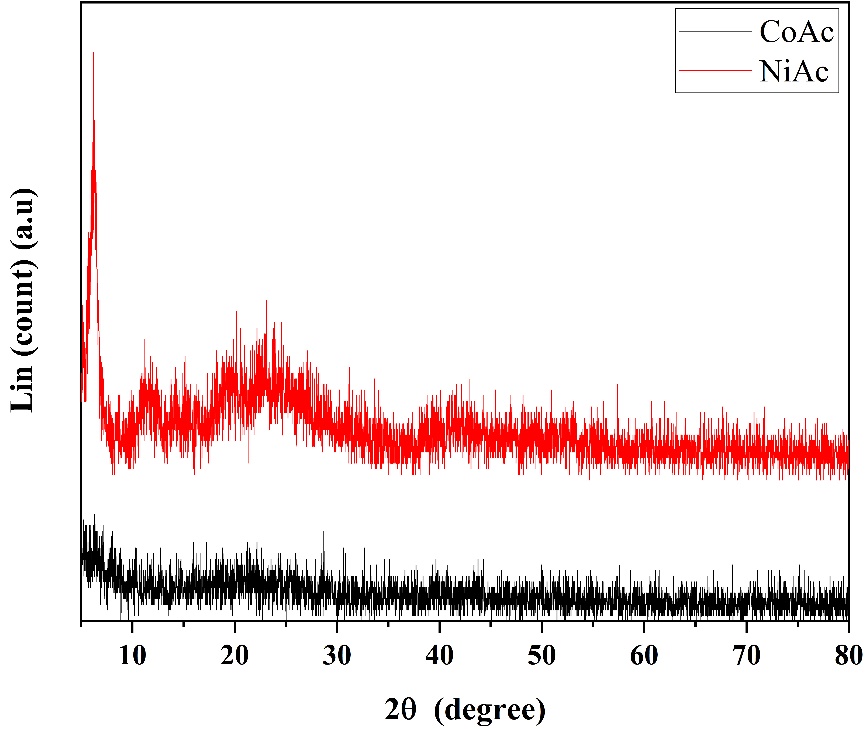
**

**Figure S10.** The powder XRD pattern of the Co(II) and Ni(II) complexes.

**Table S1**. DFT calculated dihedral angles of the ligand and metal complexes (°).

| **Angle** | **HAAT** | **Angle** | **Co(II)** |
| --- | --- | --- | --- |
| N(18)-C(19)-C(21)-C(20) | -124.286 | N(17)-C(25)-C(26)-C(27) | -133.604 |
| C(16)-N(18)-C(19)-C(21) | -121.774 | Co(19)-O(23)-C(28)-C(29) | -51.435 |
| H(23)-N(18)-C(19)-C(21) | 56.799 | H(50)-O(23)-C(28)-C(29) | 67.04 |
| N(14)-C(16)-N(18)-C(19) | 179.444 | O(21)-C(20)-O(22)-Co(19) | 4.731 |
| N(14)-C(16)-N(18)-H(23) | 0.791 | C(24)-C(20)-O(22)-Co(19) | -174.89 |
| S(17)-C(16)-N(18)-C(19) | -0.653 | O(22)-C(20)-O(21)-Co(19) | -4.766 |
| S(17)-C(16)-N(18)-H(23) | -179.305 | C(24)-C(20)-O(21)-Co(19) | 174.856 |
| N(15)-N(14)-C(16)-S(17) | -179.632 | O(13)-Co(19)-O(23)-C(28) | 172.702 |
| N(15)-N(14)-C(16)-N(18) | 0.281 | O(13)-Co(19)-O(23)-H(50) | 52.545 |
| H(22)-N(14)-C(16)-S(17) | 0.098 | N(15)-Co(19)-O(23)-C(28) | -154.88 |
| H(22)-N(14)-C(16)-N(18) | -179.988 | N(15)-Co(19)-O(23)-H(50) | 84.963 |
| C(16)-N(14)-N(15)-C(10) | 179.899 | S(18)-Co(19)-O(23)-C(28) | 76.687 |
| H(22)-N(14)-N(15)-C(10) | 0.194 | S(18)-Co(19)-O(23)-H(50) | -43.47 |
| C(9)-C(10)-N(15)-N(14) | -179.72 | O(21)-Co(19)-O(23)-C(28) | -21.489 |
| C(11)-C(10)-N(15)-N(14) | 0.086 | O(21)-Co(19)-O(23)-H(50) | -141.646 |
| C(9)-C(10)-C(11)-C(3) | 0.054 | O(22)-Co(19)-O(23)-C(28) | -88.872 |
| C(9)-C(10)-C(11)-C(6) | -179.899 | O(22)-Co(19)-O(23)-H(50) | 150.971 |
| N(15)-C(10)-C(11)-C(3) | -179.767 | O(13)-Co(19)-O(22)-C(20) | 172.998 |
| N(15)-C(10)-C(11)-C(6) | 0.28 | N(15)-Co(19)-O(22)-C(20) | -94.545 |
| C(10)-C(9)-C(12)-C(2) | -179.913 | S(18)-Co(19)-O(22)-C(20) | -12.715 |
| C(10)-C(9)-C(12)-C(3) | 0.044 | O(21)-Co(19)-O(22)-C(20) | -3.215 |
| O(13)-C(9)-C(12)-C(2) | -0.007 | O(23)-Co(19)-O(22)-C(20) | 92.082 |
| O(13)-C(9)-C(12)-C(3) | 179.951 | O(13)-Co(19)-O(21)-C(20) | -11.458 |
| C(12)-C(9)-C(10)-C(11) | -0.061 | N(15)-Co(19)-O(21)-C(20) | 93.634 |
| C(12)-C(9)-C(10)-N(15) | 179.789 | S(18)-Co(19)-O(21)-C(20) | -179.241 |
| O(13)-C(9)-C(10)-C(11) | -179.969 | O(22)-Co(19)-O(21)-C(20) | 3.199 |
| O(13)-C(9)-C(10)-N(15) | -0.119 | O(23)-Co(19)-O(21)-C(20) | -81.097 |
| C(6)-C(7)-C(8)-C(4) | -0.007 | C(16)-S(18)-Co(19)-O(13) | 115.841 |
| C(7)-C(6)-C(11)-C(3) | 0.068 | C(16)-S(18)-Co(19)-N(15) | 23.9 |
| C(7)-C(6)-C(11)-C(10) | -179.983 | C(16)-S(18)-Co(19)-O(21) | -67.32 |
| C(11)-C(6)-C(7)-C(8) | -0.033 | C(16)-S(18)-Co(19)-O(22) | -58.456 |
| C(3)-C(4)-C(8)-C(7) | 0.007 | C(16)-S(18)-Co(19)-O(23) | -161.786 |
| C(5)-C(4)-C(8)-C(7) | -179.993 | C(16)-N(17)-C(25)-C(26) | 178.611 |
| C(3)-C(4)-C(5)-C(1) | 0.02 | H(36)-N(17)-C(25)-C(26) | 34.717 |
| C(8)-C(4)-C(5)-C(1) | -179.979 | N(14)-C(16)-S(18)-Co(19) | -21.728 |
| C(4)-C(3)-C(12)-C(2) | -0.041 | N(17)-C(16)-S(18)-Co(19) | 164.889 |
| C(4)-C(3)-C(12)-C(9) | 179.995 | N(14)-C(16)-N(17)-C(25) | 18.74 |
| C(11)-C(3)-C(12)-C(2) | 179.951 | N(14)-C(16)-N(17)-H(36) | 162.885 |
| C(11)-C(3)-C(12)-C(9) | -0.012 | S(18)-C(16)-N(17)-C(25) | -167.631 |
| C(4)-C(3)-C(11)-C(6) | -0.07 | S(18)-C(16)-N(17)-H(36) | -23.487 |
| C(4)-C(3)-C(11)-C(10) | 179.966 | C(10)-N(15)-Co(19)-O(13) | 15.185 |
| C(12)-C(3)-C(11)-C(6) | 179.937 | C(10)-N(15)-Co(19)-S(18) | 112.093 |
| C(12)-C(3)-C(11)-C(10) | -0.027 | C(10)-N(15)-Co(19)-O(21) | -150.342 |
| C(11)-C(3)-C(4)-C(5) | -179.968 | C(10)-N(15)-Co(19)-O(22) | -82.588 |
| C(11)-C(3)-C(4)-C(8) | 0.032 | C(10)-N(15)-Co(19)-O(23) | -16.87 |
| C(12)-C(3)-C(4)-C(5) | 0.024 | N(14)-N(15)-Co(19)-O(13) | -127.694 |
| C(12)-C(3)-C(4)-C(8) | -179.976 | N(14)-N(15)-Co(19)-S(18) | -30.785 |
| C(1)-C(2)-C(12)-C(3) | 0.013 | N(14)-N(15)-Co(19)-O(21) | 66.78 |
| C(1)-C(2)-C(12)-C(9) | 179.966 | N(14)-N(15)-Co(19)-O(22) | 134.534 |
| C(2)-C(1)-C(5)-C(4) | -0.049 | N(14)-N(15)-Co(19)-O(23) | -159.749 |
| C(5)-C(1)-C(2)-C(12) | 0.031 | N(15)-N(14)-C(16)-N(17) | 177.745 |
|  |  | N(15)-N(14)-C(16)-S(18) | 4.25 |
|  |  | C(16)-N(14)-N(15)-C(10) | -112.427 |
|  |  | C(16)-N(14)-N(15)-Co(19) | 22.73 |
|  |  | C(9)-O(13)-Co(19)-N(15) | -19.043 |
|  |  | C(9)-O(13)-Co(19)-S(18) | -106.237 |
|  |  | C(9)-O(13)-Co(19)-O(21) | 85.963 |
|  |  | C(9)-O(13)-Co(19)-O(22) | 72.294 |
|  |  | C(9)-O(13)-Co(19)-O(23) | 157.071 |
|  |  | C(9)-C(10)-N(15)-N(14) | 131.973 |
|  |  | C(9)-C(10)-N(15)-Co(19) | -8.081 |
|  |  | C(11)-C(10)-N(15)-N(14) | -46.015 |
|  |  | C(11)-C(10)-N(15)-Co(19) | 173.931 |
|  |  | C(9)-C(10)-C(11)-C(3) | 2.832 |
|  |  | C(9)-C(10)-C(11)-C(6) | -177.732 |
|  |  | N(15)-C(10)-C(11)-C(3) | -179.09 |
|  |  | N(15)-C(10)-C(11)-C(6) | 0.345 |
|  |  | C(10)-C(9)-O(13)-Co(19) | 19.268 |
|  |  | C(12)-C(9)-O(13)-Co(19) | -169.464 |
|  |  | C(10)-C(9)-C(12)-C(2) | -179.099 |
|  |  | C(10)-C(9)-C(12)-C(3) | 2.068 |
|  |  | O(13)-C(9)-C(12)-C(2) | 8.736 |
|  |  | O(13)-C(9)-C(12)-C(3) | -170.097 |
|  |  | C(12)-C(9)-C(10)-C(11) | -3.114 |
|  |  | C(12)-C(9)-C(10)-N(15) | 178.369 |
|  |  | O(13)-C(9)-C(10)-C(11) | 169.792 |
|  |  | O(13)-C(9)-C(10)-N(15) | -8.725 |
|  |  | C(6)-C(7)-C(8)-C(4) | -0.128 |
|  |  | C(7)-C(6)-C(11)-C(3) | 0.448 |
|  |  | C(7)-C(6)-C(11)-C(10) | -178.933 |
|  |  | C(11)-C(6)-C(7)-C(8) | -0.256 |
|  |  | C(3)-C(4)-C(8)-C(7) | 0.286 |
|  |  | C(5)-C(4)-C(8)-C(7) | -179.305 |
|  |  | C(3)-C(4)-C(5)-C(1) | -0.353 |
|  |  | C(8)-C(4)-C(5)-C(1) | 179.239 |
|  |  | C(4)-C(3)-C(12)-C(2) | -0.203 |
|  |  | C(4)-C(3)-C(12)-C(9) | 178.876 |
|  |  | C(11)-C(3)-C(12)-C(2) | -179.377 |
|  |  | C(11)-C(3)-C(12)-C(9) | -0.297 |
|  |  | C(4)-C(3)-C(11)-C(6) | -0.294 |
|  |  | C(4)-C(3)-C(11)-C(10) | 179.262 |
|  |  | C(12)-C(3)-C(11)-C(6) | 178.878 |
|  |  | C(12)-C(3)-C(11)-C(10) | -1.565 |
|  |  | C(11)-C(3)-C(4)-C(5) | 179.558 |
|  |  | C(11)-C(3)-C(4)-C(8) | -0.082 |
|  |  | C(12)-C(3)-C(4)-C(5) | 0.481 |
|  |  | C(12)-C(3)-C(4)-C(8) | -179.158 |
|  |  | C(1)-C(2)-C(12)-C(3) | -0.207 |
|  |  | C(1)-C(2)-C(12)-C(9) | -178.923 |
|  |  | C(2)-C(1)-C(5)-C(4) | -0.038 |
|  |  | C(5)-C(1)-C(2)-C(12) | 0.329 |
| **Angle** | **Ni(II)** | **Angle** | **Zn(II)** |
| C(29)-C(28)-O(31)-Ni(19) | 64.344 | C(28)-C(27)-O(30)-Zn(26) | 178.876 |
| O(30)-C(28)-O(31)-Ni(19) | -123.436 | O(29)-C(27)-O(30)-Zn(26) | 0.604 |
| C(25)-C(24)-O(27)-Ni(19) | 179.344 | C(28)-C(27)-O(29)-Zn(26) | -178.883 |
| O(26)-C(24)-O(27)-Ni(19) | -1.717 | O(30)-C(27)-O(29)-Zn(26) | -0.608 |
| N(17)-C(21)-C(22)-C(23) | 118.569 | O(13)-Zn(26)-O(30)-C(27) | 111.549 |
| O(13)-Ni(19)-O(31)-C(28) | 30.376 | N(15)-Zn(26)-O(30)-C(27) | 33.104 |
| N(15)-Ni(19)-O(31)-C(28) | -64.59 | O(24)-Zn(26)-O(30)-C(27) | -97.746 |
| S(18)-Ni(19)-O(31)-C(28) | -151.816 | O(25)-Zn(26)-O(30)-C(27) | -158.384 |
| O(20)-Ni(19)-O(31)-C(28) | 120.132 | O(29)-Zn(26)-O(30)-C(27) | -0.436 |
| O(27)-Ni(19)-O(31)-C(28) | 154.424 | O(13)-Zn(26)-O(29)-C(27) | -77.55 |
| O(13)-Ni(19)-O(27)-C(24) | 50.605 | N(15)-Zn(26)-O(29)-C(27) | -162.31 |
| N(15)-Ni(19)-O(27)-C(24) | 144.426 | O(24)-Zn(26)-O(29)-C(27) | 88.069 |
| S(18)-Ni(19)-O(27)-C(24) | -126.904 | O(25)-Zn(26)-O(29)-C(27) | 63.572 |
| O(20)-Ni(19)-O(27)-C(24) | -40.658 | O(30)-Zn(26)-O(29)-C(27) | 0.48 |
| O(31)-Ni(19)-O(27)-C(24) | -73.201 | C(22)-O(25)-Zn(26)-O(13) | 170.821 |
| O(13)-Ni(19)-O(20)-H(40) | 105.07 | C(22)-O(25)-Zn(26)-N(15) | -104.427 |
| O(13)-Ni(19)-O(20)-H(52) | -16 | C(22)-O(25)-Zn(26)-O(24) | -0.602 |
| N(15)-Ni(19)-O(20)-H(40) | -28.546 | C(22)-O(25)-Zn(26)-O(29) | 27.323 |
| N(15)-Ni(19)-O(20)-H(52) | -149.617 | C(22)-O(25)-Zn(26)-O(30) | 81.936 |
| S(18)-Ni(19)-O(20)-H(40) | -73.709 | C(22)-O(24)-Zn(26)-O(13) | -17.088 |
| S(18)-Ni(19)-O(20)-H(52) | 165.221 | C(22)-O(24)-Zn(26)-N(15) | 98.558 |
| O(27)-Ni(19)-O(20)-H(40) | -160.233 | C(22)-O(24)-Zn(26)-O(25) | 0.663 |
| O(27)-Ni(19)-O(20)-H(52) | 78.697 | C(22)-O(24)-Zn(26)-O(29) | -168.326 |
| O(31)-Ni(19)-O(20)-H(40) | 13.736 | C(22)-O(24)-Zn(26)-O(30) | -106.872 |
| O(31)-Ni(19)-O(20)-H(52) | -107.335 | C(23)-C(22)-O(25)-Zn(26) | 177.931 |
| C(16)-S(18)-Ni(19)-O(13) | 122.459 | O(24)-C(22)-O(25)-Zn(26) | 0.834 |
| C(16)-S(18)-Ni(19)-N(15) | -29.315 | C(23)-C(22)-O(24)-Zn(26) | -177.942 |
| C(16)-S(18)-Ni(19)-O(20) | 145.902 | O(25)-C(22)-O(24)-Zn(26) | -0.838 |
| C(16)-S(18)-Ni(19)-O(27) | -111.501 | N(17)-C(19)-C(20)-C(21) | 126.106 |
| C(16)-S(18)-Ni(19)-O(31) | 76.957 | C(16)-N(17)-C(19)-C(20) | -86.087 |
| C(16)-N(17)-C(21)-C(22) | -81.247 | H(38)-N(17)-C(19)-C(20) | 83.529 |
| H(39)-N(17)-C(21)-C(22) | 56.869 | N(14)-C(16)-N(17)-C(19) | 0.272 |
| N(14)-C(16)-S(18)-Ni(19) | 11.297 | N(14)-C(16)-N(17)-H(38) | -169.285 |
| N(17)-C(16)-S(18)-Ni(19) | -172.583 | S(18)-C(16)-N(17)-C(19) | 169.101 |
| N(14)-C(16)-N(17)-C(21) | -25.542 | S(18)-C(16)-N(17)-H(38) | -0.455 |
| N(14)-C(16)-N(17)-H(39) | -164.027 | C(10)-N(15)-Zn(26)-O(13) | -0.605 |
| S(18)-C(16)-N(17)-C(21) | 158.324 | C(10)-N(15)-Zn(26)-O(24) | -154.323 |
| S(18)-C(16)-N(17)-H(39) | 19.839 | C(10)-N(15)-Zn(26)-O(25) | -89.548 |
| C(10)-N(15)-Ni(19)-O(13) | -14.947 | C(10)-N(15)-Zn(26)-O(29) | 108.031 |
| C(10)-N(15)-Ni(19)-S(18) | 163.596 | C(10)-N(15)-Zn(26)-O(30) | 78.973 |
| C(10)-N(15)-Ni(19)-O(20) | 118.492 | N(14)-N(15)-Zn(26)-O(13) | -177.667 |
| C(10)-N(15)-Ni(19)-O(27) | -108.925 | N(14)-N(15)-Zn(26)-O(24) | 28.616 |
| C(10)-N(15)-Ni(19)-O(31) | 77.723 | N(14)-N(15)-Zn(26)-O(25) | 93.391 |
| N(14)-N(15)-Ni(19)-O(13) | -136.053 | N(14)-N(15)-Zn(26)-O(29) | -69.03 |
| N(14)-N(15)-Ni(19)-S(18) | 42.491 | N(14)-N(15)-Zn(26)-O(30) | -98.088 |
| N(14)-N(15)-Ni(19)-O(20) | -2.613 | N(15)-N(14)-C(16)-N(17) | -72.314 |
| N(14)-N(15)-Ni(19)-O(27) | 129.97 | N(15)-N(14)-C(16)-S(18) | 118.774 |
| N(14)-N(15)-Ni(19)-O(31) | -43.383 | H(37)-N(14)-C(16)-N(17) | 122.389 |
| N(15)-N(14)-C(16)-N(17) | -156.262 | H(37)-N(14)-C(16)-S(18) | -46.522 |
| N(15)-N(14)-C(16)-S(18) | 19.934 | C(16)-N(14)-N(15)-C(10) | 154.409 |
| H(38)-N(14)-C(16)-N(17) | -2.242 | C(16)-N(14)-N(15)-Zn(26) | -28.98 |
| H(38)-N(14)-C(16)-S(18) | 173.954 | H(37)-N(14)-N(15)-C(10) | -40.356 |
| C(16)-N(14)-N(15)-C(10) | -158.321 | H(37)-N(14)-N(15)-Zn(26) | 136.255 |
| C(16)-N(14)-N(15)-Ni(19) | -46.859 | C(9)-O(13)-Zn(26)-N(15) | 0.235 |
| H(38)-N(14)-N(15)-C(10) | 47.215 | C(9)-O(13)-Zn(26)-O(24) | 121.607 |
| H(38)-N(14)-N(15)-Ni(19) | 158.677 | C(9)-O(13)-Zn(26)-O(25) | 106.067 |
| C(9)-O(13)-Ni(19)-N(15) | 12.434 | C(9)-O(13)-Zn(26)-O(29) | -88.653 |
| C(9)-O(13)-Ni(19)-S(18) | -139.263 | C(9)-O(13)-Zn(26)-O(30) | -147.979 |
| C(9)-O(13)-Ni(19)-O(20) | -162.67 | C(9)-C(10)-N(15)-N(14) | 177.875 |
| C(9)-O(13)-Ni(19)-O(27) | 94.854 | C(9)-C(10)-N(15)-Zn(26) | 0.824 |
| C(9)-O(13)-Ni(19)-O(31) | -93.853 | C(11)-C(10)-N(15)-N(14) | -2.605 |
| C(9)-C(10)-N(15)-N(14) | 127.3 | C(11)-C(10)-N(15)-Zn(26) | -179.656 |
| C(9)-C(10)-N(15)-Ni(19) | 14.34 | C(9)-C(10)-C(11)-C(3) | 0.151 |
| C(11)-C(10)-N(15)-N(14) | -65.028 | C(9)-C(10)-C(11)-C(6) | 179.452 |
| C(11)-C(10)-N(15)-Ni(19) | -177.988 | N(15)-C(10)-C(11)-C(3) | -179.39 |
| C(9)-C(10)-C(11)-C(3) | -1.4 | N(15)-C(10)-C(11)-C(6) | -0.09 |
| C(9)-C(10)-C(11)-C(6) | 179.062 | C(10)-C(9)-O(13)-Zn(26) | 0.154 |
| N(15)-C(10)-C(11)-C(3) | -169.644 | C(12)-C(9)-O(13)-Zn(26) | 179.904 |
| N(15)-C(10)-C(11)-C(6) | 10.817 | C(10)-C(9)-C(12)-C(2) | -179.691 |
| C(10)-C(9)-O(13)-Ni(19) | -6.513 | C(10)-C(9)-C(12)-C(3) | 0.096 |
| C(12)-C(9)-O(13)-Ni(19) | 176.381 | O(13)-C(9)-C(12)-C(2) | 0.542 |
| C(10)-C(9)-C(12)-C(2) | 179.659 | O(13)-C(9)-C(12)-C(3) | -179.672 |
| C(10)-C(9)-C(12)-C(3) | -0.663 | C(12)-C(9)-C(10)-C(11) | -0.157 |
| O(13)-C(9)-C(12)-C(2) | -2.948 | C(12)-C(9)-C(10)-N(15) | 179.49 |
| O(13)-C(9)-C(12)-C(3) | 176.73 | O(13)-C(9)-C(10)-C(11) | 179.65 |
| C(12)-C(9)-C(10)-C(11) | 1.311 | O(13)-C(9)-C(10)-N(15) | -0.703 |
| C(12)-C(9)-C(10)-N(15) | 171.808 | C(6)-C(7)-C(8)-C(4) | 0.051 |
| O(13)-C(9)-C(10)-C(11) | -176.299 | C(7)-C(6)-C(11)-C(3) | -0.168 |
| O(13)-C(9)-C(10)-N(15) | -5.803 | C(7)-C(6)-C(11)-C(10) | -179.392 |
| C(6)-C(7)-C(8)-C(4) | 0.196 | C(11)-C(6)-C(7)-C(8) | 0.093 |
| C(7)-C(6)-C(11)-C(3) | -0.069 | C(3)-C(4)-C(8)-C(7) | -0.112 |
| C(7)-C(6)-C(11)-C(10) | 179.426 | C(5)-C(4)-C(8)-C(7) | 179.717 |
| C(11)-C(6)-C(7)-C(8) | -0.063 | C(3)-C(4)-C(5)-C(1) | 0.099 |
| C(3)-C(4)-C(8)-C(7) | -0.183 | C(8)-C(4)-C(5)-C(1) | -179.73 |
| C(5)-C(4)-C(8)-C(7) | 179.441 | C(4)-C(3)-C(12)-C(2) | 0.188 |
| C(3)-C(4)-C(5)-C(1) | 0.184 | C(4)-C(3)-C(12)-C(9) | -179.636 |
| C(8)-C(4)-C(5)-C(1) | -179.44 | C(11)-C(3)-C(12)-C(2) | 179.823 |
| C(4)-C(3)-C(12)-C(2) | 0.072 | C(11)-C(3)-C(12)-C(9) | 0 |
| C(4)-C(3)-C(12)-C(9) | -179.677 | C(4)-C(3)-C(11)-C(6) | 0.11 |
| C(11)-C(3)-C(12)-C(2) | 179.513 | C(4)-C(3)-C(11)-C(10) | 179.54 |
| C(11)-C(3)-C(12)-C(9) | -0.236 | C(12)-C(3)-C(11)-C(6) | -179.524 |
| C(4)-C(3)-C(11)-C(6) | 0.073 | C(12)-C(3)-C(11)-C(10) | -0.094 |
| C(4)-C(3)-C(11)-C(10) | -179.571 | C(11)-C(3)-C(4)-C(5) | -179.812 |
| C(12)-C(3)-C(11)-C(6) | -179.366 | C(11)-C(3)-C(4)-C(8) | 0.035 |
| C(12)-C(3)-C(11)-C(10) | 0.99 | C(12)-C(3)-C(4)-C(5) | -0.223 |
| C(11)-C(3)-C(4)-C(5) | -179.616 | C(12)-C(3)-C(4)-C(8) | 179.624 |
| C(11)-C(3)-C(4)-C(8) | 0.053 | C(1)-C(2)-C(12)-C(3) | -0.016 |
| C(12)-C(3)-C(4)-C(5) | -0.244 | C(1)-C(2)-C(12)-C(9) | 179.746 |
| C(12)-C(3)-C(4)-C(8) | 179.425 | C(2)-C(1)-C(5)-C(4) | 0.059 |
| C(1)-C(2)-C(12)-C(3) | 0.159 | C(5)-C(1)-C(2)-C(12) | -0.099 |
| C(1)-C(2)-C(12)-C(9) | 179.804 |  |  |
| C(2)-C(1)-C(5)-C(4) | 0.039 |  |  |
| C(5)-C(1)-C(2)-C(12) | -0.218 |  |  |

**Table S2**. DFT calculated bond length of the ligand and metal complexes (Å).

| **Bond** | **HAAT** | **Bond** | **Co(II)** | **Bond** | **Ni(II)** | **Bond** | **Zn(II)** |
| --- | --- | --- | --- | --- | --- | --- | --- |
| C(20)-C(21) | 1.327 | C(28)-C(29) | 1.512 | C(28)-O(31) | 1.341 | C(27)-O(30) | 1.335 |
| C(19)-C(21) | 1.482 | C(26)-C(27) | 1.328 | C(28)-O(30) | 1.223 | C(27)-O(29) | 1.226 |
| N(18)-H(23) | 1.031 | C(25)-C(26) | 1.492 | C(28)-C(29) | 1.502 | C(27)-C(28) | 1.493 |
| N(18)-C(19) | 1.435 | O(23)-H(50) | 0.963 | C(24)-O(27) | 1.323 | Zn(26)-O(30) | 1.9 |
| C(16)-N(18) | 1.331 | O(23)-C(28) | 1.447 | C(24)-O(26) | 1.239 | Zn(26)-O(29) | 1.909 |
| C(16)-S(17) | 1.658 | C(20)-C(24) | 1.481 | C(24)-C(25) | 1.489 | O(25)-Zn(26) | 1.898 |
| N(14)-H(22) | 1.028 | C(20)-O(22) | 1.304 | C(22)-C(23) | 1.328 | O(24)-Zn(26) | 1.909 |
| N(14)-C(16) | 1.385 | C(20)-O(21) | 1.31 | C(21)-C(22) | 1.494 | C(22)-O(25) | 1.335 |
| N(14)-N(15) | 1.31 | Co(19)-O(23) | 2.041 | O(20)-H(52) | 0.965 | C(22)-O(24) | 1.226 |
| C(10)-N(15) | 1.291 | Co(19)-O(22) | 1.948 | O(20)-H(40) | 0.978 | C(22)-C(23) | 1.493 |
| C(10)-C(11) | 1.452 | Co(19)-O(21) | 1.934 | Ni(19)-O(31) | 1.858 | C(20)-C(21) | 1.343 |
| C(9)-O(13) | 1.212 | S(18)-Co(19) | 2.244 | Ni(19)-O(27) | 1.856 | C(19)-C(20) | 1.508 |
| C(9)-C(12) | 1.476 | N(17)-H(36) | 0.996 | Ni(19)-O(20) | 1.926 | N(17)-H(38) | 0.999 |
| C(9)-C(10) | 1.502 | N(17)-C(25) | 1.484 | S(18)-Ni(19) | 2.3 | N(17)-C(19) | 1.456 |
| C(7)-C(8) | 1.375 | C(16)-S(18) | 1.795 | N(17)-H(39) | 0.998 | C(16)-S(18) | 1.692 |
| C(6)-C(11) | 1.379 | C(16)-N(17) | 1.39 | N(17)-C(21) | 1.484 | C(16)-N(17) | 1.419 |
| C(6)-C(7) | 1.41 | N(15)-Co(19) | 1.862 | C(16)-S(18) | 1.719 | N(15)-Zn(26) | 1.949 |
| C(4)-C(8) | 1.411 | N(14)-C(16) | 1.343 | C(16)-N(17) | 1.411 | N(14)-H(37) | 0.998 |
| C(4)-C(5) | 1.409 | N(14)-N(15) | 1.393 | N(15)-Ni(19) | 1.852 | N(14)-C(16) | 1.412 |
| C(3)-C(12) | 1.4 | O(13)-Co(19) | 1.916 | N(14)-H(38) | 1.005 | N(14)-N(15) | 1.355 |
| C(3)-C(11) | 1.417 | C(10)-N(15) | 1.376 | N(14)-C(16) | 1.365 | O(13)-Zn(26) | 1.898 |
| C(3)-C(4) | 1.399 | C(10)-C(11) | 1.448 | N(14)-N(15) | 1.475 | C(10)-N(15) | 1.282 |
| C(2)-C(12) | 1.371 | C(9)-O(13) | 1.295 | O(13)-Ni(19) | 1.854 | C(10)-C(11) | 1.466 |
| C(1)-C(5) | 1.38 | C(9)-C(12) | 1.462 | C(10)-N(15) | 1.414 | C(9)-O(13) | 1.228 |
| C(1)-C(2) | 1.406 | C(9)-C(10) | 1.449 | C(10)-C(11) | 1.455 | C(9)-C(12) | 1.461 |
|  |  | C(7)-C(8) | 1.374 | C(9)-O(13) | 1.32 | C(9)-C(10) | 1.465 |
|  |  | C(6)-C(11) | 1.37 | C(9)-C(12) | 1.473 | C(7)-C(8) | 1.354 |
|  |  | C(6)-C(7) | 1.423 | C(9)-C(10) | 1.395 | C(6)-C(11) | 1.341 |
|  |  | C(4)-C(8) | 1.425 | C(7)-C(8) | 1.374 | C(6)-C(7) | 1.485 |
|  |  | C(4)-C(5) | 1.424 | C(6)-C(11) | 1.368 | C(4)-C(8) | 1.474 |
|  |  | C(3)-C(12) | 1.433 | C(6)-C(7) | 1.423 | C(4)-C(5) | 1.475 |
|  |  | C(3)-C(11) | 1.437 | C(4)-C(8) | 1.424 | C(3)-C(12) | 1.454 |
|  |  | C(3)-C(4) | 1.386 | C(4)-C(5) | 1.424 | C(3)-C(11) | 1.455 |
|  |  | C(2)-C(12) | 1.369 | C(3)-C(12) | 1.432 | C(3)-C(4) | 1.335 |
|  |  | C(1)-C(5) | 1.375 | C(3)-C(11) | 1.437 | C(2)-C(12) | 1.34 |
|  |  | C(1)-C(2) | 1.422 | C(3)-C(4) | 1.387 | C(1)-C(5) | 1.354 |
|  |  |  |  | C(2)-C(12) | 1.366 | C(1)-C(2) | 1.485 |
|  |  |  |  | C(1)-C(5) | 1.374 |  |  |
|  |  |  |  | C(1)-C(2) | 1.424 |  |  |

**Table S3**. DFT calculated bond angles of the ligand and metal complexes (°).

| **Angle** | **HAAT** | **Angle** | **Co(II)** |
| --- | --- | --- | --- |
| C(20)-C(21)-C(19) | 123.818 | C(29)-C(28)-O(23) | 113.585 |
| C(21)-C(19)-N(18) | 111.744 | C(27)-C(26)-C(25) | 122.426 |
| H(23)-N(18)-C(19) | 121.06 | C(26)-C(25)-N(17) | 110.028 |
| H(23)-N(18)-C(16) | 114.888 | H(50)-O(23)-C(28) | 104.117 |
| C(19)-N(18)-C(16) | 124.038 | H(50)-O(23)-Co(19) | 99.626 |
| N(18)-C(16)-S(17) | 128.067 | C(28)-O(23)-Co(19) | 133.533 |
| N(18)-C(16)-N(14) | 113.343 | C(20)-O(22)-Co(19) | 89.916 |
| S(17)-C(16)-N(14) | 118.591 | C(20)-O(21)-Co(19) | 90.356 |
| N(14)-N(15)-C(10) | 120.566 | C(24)-C(20)-O(22) | 124.295 |
| H(22)-N(14)-C(16) | 115.238 | C(24)-C(20)-O(21) | 123.978 |
| H(22)-N(14)-N(15) | 124.373 | O(22)-C(20)-O(21) | 111.726 |
| C(16)-N(14)-N(15) | 120.388 | O(23)-Co(19)-O(22) | 86.015 |
| C(9)-C(12)-C(3) | 107.459 | O(23)-Co(19)-O(21) | 93.377 |
| C(9)-C(12)-C(2) | 132.614 | O(23)-Co(19)-S(18) | 97.604 |
| C(3)-C(12)-C(2) | 119.927 | O(23)-Co(19)-N(15) | 172.743 |
| C(10)-C(11)-C(6) | 136.394 | O(23)-Co(19)-O(13) | 81.562 |
| C(10)-C(11)-C(3) | 106.002 | O(22)-Co(19)-O(21) | 67.736 |
| C(6)-C(11)-C(3) | 117.604 | O(22)-Co(19)-S(18) | 165.187 |
| N(15)-C(10)-C(11) | 132.752 | O(22)-Co(19)-N(15) | 91.014 |
| N(15)-C(10)-C(9) | 118.789 | O(22)-Co(19)-O(13) | 97.724 |
| C(11)-C(10)-C(9) | 108.459 | O(21)-Co(19)-S(18) | 97.638 |
| O(13)-C(9)-C(12) | 128.269 | O(21)-Co(19)-N(15) | 91.615 |
| O(13)-C(9)-C(10) | 126.591 | O(21)-Co(19)-O(13) | 165.01 |
| C(12)-C(9)-C(10) | 105.14 | S(18)-Co(19)-N(15) | 86.937 |
| C(7)-C(8)-C(4) | 119.964 | S(18)-Co(19)-O(13) | 97.017 |
| C(8)-C(7)-C(6) | 122.644 | N(15)-Co(19)-O(13) | 92.298 |
| C(11)-C(6)-C(7) | 119.189 | Co(19)-S(18)-C(16) | 90.092 |
| C(4)-C(5)-C(1) | 120.776 | H(36)-N(17)-C(25) | 114.978 |
| C(8)-C(4)-C(5) | 127.322 | H(36)-N(17)-C(16) | 114.223 |
| C(8)-C(4)-C(3) | 116.519 | C(25)-N(17)-C(16) | 121 |
| C(5)-C(4)-C(3) | 116.158 | S(18)-C(16)-N(17) | 119.825 |
| C(12)-C(3)-C(11) | 112.941 | S(18)-C(16)-N(14) | 121.804 |
| C(12)-C(3)-C(4) | 122.979 | N(17)-C(16)-N(14) | 118.052 |
| C(11)-C(3)-C(4) | 124.08 | Co(19)-N(15)-N(14) | 118.229 |
| C(12)-C(2)-C(1) | 118.163 | Co(19)-N(15)-C(10) | 104.611 |
| C(5)-C(1)-C(2) | 121.997 | N(14)-N(15)-C(10) | 124.09 |
|  |  | C(16)-N(14)-N(15) | 112.679 |
|  |  | Co(19)-O(13)-C(9) | 101.782 |
|  |  | C(9)-C(12)-C(3) | 105.076 |
|  |  | C(9)-C(12)-C(2) | 136.224 |
|  |  | C(3)-C(12)-C(2) | 118.691 |
|  |  | C(10)-C(11)-C(6) | 136.252 |
|  |  | C(10)-C(11)-C(3) | 105.37 |
|  |  | C(6)-C(11)-C(3) | 118.377 |
|  |  | N(15)-C(10)-C(11) | 135.792 |
|  |  | N(15)-C(10)-C(9) | 115.298 |
|  |  | C(11)-C(10)-C(9) | 108.887 |
|  |  | O(13)-C(9)-C(12) | 129.499 |
|  |  | O(13)-C(9)-C(10) | 121.594 |
|  |  | C(12)-C(9)-C(10) | 108.446 |
|  |  | C(7)-C(8)-C(4) | 120.059 |
|  |  | C(8)-C(7)-C(6) | 123.066 |
|  |  | C(11)-C(6)-C(7) | 118.249 |
|  |  | C(4)-C(5)-C(1) | 120.239 |
|  |  | C(8)-C(4)-C(5) | 127.564 |
|  |  | C(8)-C(4)-C(3) | 116.245 |
|  |  | C(5)-C(4)-C(3) | 116.19 |
|  |  | C(12)-C(3)-C(11) | 112.13 |
|  |  | C(12)-C(3)-C(4) | 123.861 |
|  |  | C(11)-C(3)-C(4) | 124.004 |
|  |  | C(12)-C(2)-C(1) | 118.179 |
|  |  | C(5)-C(1)-C(2) | 122.838 |
| **Angle** | **Ni(II)** | **Angle** | **Zn(II)** |
| C(28)-O(31)-Ni(19) | 131.849 | C(27)-O(30)-Zn(26) | 97.885 |
| O(31)-C(28)-O(30) | 109.671 | C(27)-O(29)-Zn(26) | 101.627 |
| O(31)-C(28)-C(29) | 124.369 | O(30)-C(27)-O(29) | 98.96 |
| O(30)-C(28)-C(29) | 125.455 | O(30)-C(27)-C(28) | 130.572 |
| C(24)-O(27)-Ni(19) | 103.207 | O(29)-C(27)-C(28) | 130.442 |
| O(27)-C(24)-O(26) | 111.486 | O(30)-Zn(26)-O(29) | 61.523 |
| O(27)-C(24)-C(25) | 119.832 | O(30)-Zn(26)-O(25) | 105.959 |
| O(26)-C(24)-C(25) | 128.672 | O(30)-Zn(26)-O(24) | 91.231 |
| C(23)-C(22)-C(21) | 122.737 | O(30)-Zn(26)-N(15) | 147.624 |
| C(22)-C(21)-N(17) | 112.79 | O(30)-Zn(26)-O(13) | 88.821 |
| H(52)-O(20)-H(40) | 108.276 | O(29)-Zn(26)-O(25) | 156.121 |
| H(52)-O(20)-Ni(19) | 115.531 | O(29)-Zn(26)-O(24) | 97.011 |
| H(40)-O(20)-Ni(19) | 107.598 | O(29)-Zn(26)-N(15) | 90.594 |
| O(31)-Ni(19)-O(27) | 169.505 | O(29)-Zn(26)-O(13) | 108.614 |
| O(31)-Ni(19)-O(20) | 68.817 | O(25)-Zn(26)-O(24) | 61.496 |
| O(31)-Ni(19)-S(18) | 86.508 | O(25)-Zn(26)-N(15) | 105.802 |
| O(31)-Ni(19)-N(15) | 106.127 | O(25)-Zn(26)-O(13) | 90.389 |
| O(31)-Ni(19)-O(13) | 91.337 | O(24)-Zn(26)-N(15) | 109.391 |
| O(27)-Ni(19)-O(20) | 102.415 | O(24)-Zn(26)-O(13) | 150.712 |
| O(27)-Ni(19)-S(18) | 87.272 | N(15)-Zn(26)-O(13) | 84.845 |
| O(27)-Ni(19)-N(15) | 82.116 | Zn(26)-O(25)-C(22) | 98.001 |
| O(27)-Ni(19)-O(13) | 94.532 | Zn(26)-O(24)-C(22) | 101.603 |
| O(20)-Ni(19)-S(18) | 86.925 | O(25)-C(22)-O(24) | 98.891 |
| O(20)-Ni(19)-N(15) | 173.25 | O(25)-C(22)-C(23) | 130.593 |
| O(20)-Ni(19)-O(13) | 90.256 | O(24)-C(22)-C(23) | 130.443 |
| S(18)-Ni(19)-N(15) | 88.31 | C(21)-C(20)-C(19) | 121.861 |
| S(18)-Ni(19)-O(13) | 176.928 | C(20)-C(19)-N(17) | 110.176 |
| N(15)-Ni(19)-O(13) | 94.396 | H(38)-N(17)-C(19) | 117.658 |
| Ni(19)-S(18)-C(16) | 89.568 | H(38)-N(17)-C(16) | 118.263 |
| H(39)-N(17)-C(21) | 113.564 | C(19)-N(17)-C(16) | 123.288 |
| H(39)-N(17)-C(16) | 112.59 | S(18)-C(16)-N(17) | 118.959 |
| C(21)-N(17)-C(16) | 120.579 | S(18)-C(16)-N(14) | 118.188 |
| S(18)-C(16)-N(17) | 121.137 | N(17)-C(16)-N(14) | 121.934 |
| S(18)-C(16)-N(14) | 119.548 | Zn(26)-N(15)-N(14) | 124.978 |
| N(17)-C(16)-N(14) | 119.203 | Zn(26)-N(15)-C(10) | 109.696 |
| Ni(19)-N(15)-N(14) | 104.616 | N(14)-N(15)-C(10) | 125.255 |
| Ni(19)-N(15)-C(10) | 102.032 | H(37)-N(14)-C(16) | 118.24 |
| N(14)-N(15)-C(10) | 115.869 | H(37)-N(14)-N(15) | 118.672 |
| H(38)-N(14)-C(16) | 120.1 | C(16)-N(14)-N(15) | 121.468 |
| H(38)-N(14)-N(15) | 118.456 | Zn(26)-O(13)-C(9) | 111.87 |
| C(16)-N(14)-N(15) | 116.193 | C(9)-C(12)-C(3) | 105.723 |
| Ni(19)-O(13)-C(9) | 103.015 | C(9)-C(12)-C(2) | 134.206 |
| C(9)-C(12)-C(3) | 104.362 | C(3)-C(12)-C(2) | 120.07 |
| C(9)-C(12)-C(2) | 136.887 | C(10)-C(11)-C(6) | 134.953 |
| C(3)-C(12)-C(2) | 118.751 | C(10)-C(11)-C(3) | 105.407 |
| C(10)-C(11)-C(6) | 137.108 | C(6)-C(11)-C(3) | 119.636 |
| C(10)-C(11)-C(3) | 104.814 | N(15)-C(10)-C(11) | 135.862 |
| C(6)-C(11)-C(3) | 118.077 | N(15)-C(10)-C(9) | 115.362 |
| N(15)-C(10)-C(11) | 133.286 | C(11)-C(10)-C(9) | 108.775 |
| N(15)-C(10)-C(9) | 116.069 | O(13)-C(9)-C(12) | 133.145 |
| C(11)-C(10)-C(9) | 109.724 | O(13)-C(9)-C(10) | 118.222 |
| O(13)-C(9)-C(12) | 128.811 | C(12)-C(9)-C(10) | 108.633 |
| O(13)-C(9)-C(10) | 121.785 | C(7)-C(8)-C(4) | 120.067 |
| C(12)-C(9)-C(10) | 109.353 | C(8)-C(7)-C(6) | 121.722 |
| C(7)-C(8)-C(4) | 119.972 | C(11)-C(6)-C(7) | 117.609 |
| C(8)-C(7)-C(6) | 123.081 | C(4)-C(5)-C(1) | 120.142 |
| C(11)-C(6)-C(7) | 118.442 | C(8)-C(4)-C(5) | 126.853 |
| C(4)-C(5)-C(1) | 120.216 | C(8)-C(4)-C(3) | 116.549 |
| C(8)-C(4)-C(5) | 127.794 | C(5)-C(4)-C(3) | 116.598 |
| C(8)-C(4)-C(3) | 116.172 | C(12)-C(3)-C(11) | 111.462 |
| C(5)-C(4)-C(3) | 116.033 | C(12)-C(3)-C(4) | 124.12 |
| C(12)-C(3)-C(11) | 111.728 | C(11)-C(3)-C(4) | 124.417 |
| C(12)-C(3)-C(4) | 124.013 | C(12)-C(2)-C(1) | 117.403 |
| C(11)-C(3)-C(4) | 124.257 | C(5)-C(1)-C(2) | 121.666 |
| C(12)-C(2)-C(1) | 118.053 |  |  |
| C(5)-C(1)-C(2) | 122.934 |  |  |
